# Impact of neuroinflammation on brain glutamate and dopamine signalling in schizophrenia: an update

**DOI:** 10.1007/s11011-025-01548-3

**Published:** 2025-02-05

**Authors:** Usha Nayak, Jyothsna Manikkath, Devinder Arora, Jayesh Mudgal

**Affiliations:** 1 Manipal College of Pharmaceutical Sciences, Manipal Academy of Higher Education, Manipal, 576104 Karnataka India; 2 Department of Pharmaceutics, Manipal College of Pharmaceutical Sciences, Manipal Academy of Higher Education, Manipal, 576104 Karnataka India; 3 School of Pharmacy and Medical Sciences, Griffith University, Gold Coast, QLD 4222 Australia; 4 Department of Pharmacology, Manipal College of Pharmaceutical Sciences, Manipal Academy of Higher Education, Manipal, 576104 Karnataka India

**Keywords:** Schizophrenia, Inflammation, Neurotransmitters, Kynurenic acid, Antipsychotics, Immunomodulatory drugs

## Abstract

Schizophrenia is one of the most severe and chronic psychiatric disorders. Over the years, numerous treatment options have been introduced for schizophrenia. Although they are relatively successful in managing the positive symptoms of schizophrenia, most of the current treatments have a negligible effect on the negative and cognitive symptoms. Thus, none of them could prevent the relapse of psychotic episodes. Among the numerous hypotheses explaining the development and progression of schizophrenia, the cytokine hypothesis explains the role of inflammatory markers as a significant culprit in the development of schizophrenia. Elevated cytokines are reported in animal models and schizophrenic patients. The cytokine hypothesis is based on how increased inflammatory markers can cause changes in the dopaminergic, glutamate, and tryptophan metabolism pathways, like that observed in schizophrenic patients. Reasons, such as autoimmune disease, maternal immune activation, infection, etc., can pave the way for the development of schizophrenia and are associated with the negative, positive and cognitive symptoms of schizophrenia. Thus, there is a need to focus on the significance of anti-inflammatory drugs against these symptoms. The development of new treatment strategies in the management of schizophrenia can provide better therapeutic outcomes in terms of the severity of symptoms and treatment of drug-resistant schizophrenia. This review attempts to explain the association between elevated inflammatory markers and various neurotransmitters, and the possible use of medications like nonsteroidal anti-inflammatory drugs, monoclonal antibodies, statins, and estrogens as adjuvant therapy. Over the years, these hypotheses have been the basis for drug discovery for the treatment of schizophrenia.

## Highlights

Activated microglia are involved in the pathogenesis of schizophrenia.

Inflammatory markers induce IDO and tryptophan dioxygenase to increase KYA.

Inflammatory markers influence dopamine and glutamate signalling for psychosis.

Inflammatory markers cause glutamate-mediated excitotoxicity and oxidative stress.

Anti-inflammatory drugs approach discussed for the management of schizophrenia.

## Introduction

Schizophrenia, a chronic and severe psychiatric disorder, is a clinical syndrome characterized by variable and fundamentally disruptive psychopathology of perception, cognition, emotion, and other characteristics of behaviour (McGrath et al. 2008). Approximately 1% of the total world population is affected by schizophrenia, and it has an annual incidence of 3.89 to 4.03 per 1000 subjects. Although categorized as a low prevalence disorder, its economic burden is significant and it is ranked among the top 15 causes of disability worldwide (Moreno-Küstner et al. 2018). Schizophrenia is associated with a dysregulation in serotonergic, dopaminergic, glutamatergic, and gamma-aminobutyric acid (GABA) signaling pathways, which manifests into disrupted activity of a wide range of macro- and micro-neuronal circuits and behavioural, cognitive, and social dysfunction. Various etiological factors including neuroanatomic, genetic, neurochemical, and other biological dysregulation, and *in utero* exposure to bacterial, viral or parasitic infection, are said to be associated with the risk of developing schizophrenia (McGrath et al. 2008; Brown and Derkits 2010). Heredity/ genetic risk factors are responsible for around 81% and varied environmental factors contribute approximately to 11%, respectively of the schizophrenia cases, as evidenced by meta-analysis studies (Sullivan et al. 2003).

The three primary domains of schizophrenic symptoms are negative, positive, and cognitive. Positive symptoms are characterized by hallucinations and delusions whereas negative symptoms include emotional blunting, anhedonia, avolition etc. Furthermore, cognitive deficits related to working memory and executive functions (Shah and González-Maeso 2019; Zamanpoor 2020). Even after several decades of the introduction of antipsychotic therapy, the antipsychotics in existence are less effective towards the negative and cognitive symptoms of schizophrenia, which also happen to be the critical determinants of functional disabilities. Moreover, the effectiveness of antipsychotics towards positive symptoms in all schizophrenic patients is limited (Moghaddam and Javitt 2012). Dose-dependent side effects, some of which are severe to warrant treatment discontinuation, are another limitation of antipsychotics (Muench and Hamer 2010). All these highlight the need for the development of an adjuvant therapy which can supplement the use of antipsychotic medication.

The current understanding of factors contributing to schizophrenia points towards the role of neuronal inflammation in the onset and progression of the disease. Inflammation is common to many disorders such as diabetes mellitus, hypertension, coronary artery disease and also schizophrenia. Inflammation and schizophrenia could be a likely chicken and egg conundrum, with one being a cause or consequence of the other. Therefore, the role of inflammation in the dysregulation of neurotransmitters in the brain leading to the development of the pathological circuit of schizophrenia, is a crucial area of investigation with pharmacotherapeutic implications, and is the focus of this review. Further, the present review also addresses the influence of various drugs that alter the inflammatory mediators in subsiding the positive, negative, and cognitive effects of schizophrenia.

### Microglial inflammation and psychosis

Microglial cells, also called macrophages of the brain, are immune cells ubiquitously distributed in the central nervous system (CNS) which strive to maintain homeostasis within the CNS (Fujita and Yamashita 2021). These cells constitute about 10–15% of glial cells of the brain (Salter and Stevens 2017). They are of mesodermal origin, in contrast to other parenchymal cells in the brain, which are of multiple neuroectodermal lineages. Glial cells are broadly classified as astrocytes, oligodendrocytes, ependymal and microglial cells (Tan et al. 2020). Glial cells regulate the immune system by producing both proinflammatory cytokines as well as anti-inflammatory components. They also produce neurotrophic factors and aid in neurodevelopment (Bergink et al. 2014). Microglia presents major histocompatibility complex (MHC) class I and II molecules. MHC II is expressed by antigen-presenting cells (APCs) such as dendritic cells, mononuclear phagocytes and microglia. Microglia plays a significant role in initiating an innate immune response and represent the major APCs in the brain parenchyma that express MHC II in neurodegenerative disease (Barichello et al. 2019; Nimmerjahn et al. 2005). A convincing amount of data shows an integrated relationship between psychiatric disorders and microglial cells. Increased microglial activity is a well-known risk factor for the development of mental illness (Lurie 2018). Post-mortem reports of schizophrenic patients upon evaluation showed increased density of reactive microglia in different subunits of the brain (Mattei et al. 2015). Various factors like infection in the CNS due to bacteria, viruses, and fungi, or factors like multiple sclerosis, aging, dementia, and neurotoxicity in CNS, etc. activate microglial cells. For instance, elevation of inflammatory markers due to influenza virus (Wong et al. 2018), a study conducted by Opsteen et al. 2023 reported chronic immune activation following long SARS-CoV-2 infection, wherein elevation of CD4^+^ and CD8^+^ T-cell was observed (Opsteen et al. 2023). A first case of psychosis due to mRNA-based COVID-19 vaccine was reported in 2021, where a 31-year-old male was showing erratic and bizarre behaviour with auditory hallucination and delusions after one month of mRNA-based COVID-19 vaccine administration. This could be due to an exaggerated immune response to the vaccine, which includes the release of excessive amounts of proinflammatory cytokines (Reinfeld et al. 2021). Further, the COVID-19 pandemic highlighted the connection between respiratory viral infections and the onset of psychosis, with persistent systemic inflammation associated with the development of schizophrenia (Kulaga and Miller 2021).

Activated microglial cells play a significant role in the production of cytokines and early brain development (Salter and Beggs 2014). During maternal immune activation (MIA), pro-inflammatory mediators present in the maternal blood enhance the permeability of the placenta and the fetal blood brain barrier (BBB), thereby letting inflammatory mediators enter the fetal brain. Once the inflammatory markers enter the fetal brain, they activate microglial cells, which in turn release various proinflammatory mediators like tumor necrosis factor alpha (TNF-α), interleukin (IL)−1β, and IL-6 in the brain (Barichello et al. 2019; Estes and McAllister 2016).

In an experimental setup when the maternal immune system was activated by administrating polycytidylic acid (poly I: C) into the maternal body, micro-glial markers were altered subsequently, indicating a considerable degree of microglial activation upon exposure to poly I: C (Hadar et al. 2017). In another study, Sprague Dawley rats that were put in a stressful situation were found to have a drastic increase in mature T-cells that were positive for serum proinflammatory cytokines and intracellular type-2-like cytokine. These led to microglial cell activation and increased neuronal firing in the basolateral amygdala. These events were minimized after abolishing microglial activation. Pratt et al. (2013) injected poly I: C (which mimics viral infection) into pregnant mice on embryonic day 12.5 and then collected the fetal brain on embryonic day 16.5 to check the inflammatory status of microglia. It was found that fetal microglia had expressed various inflammatory markers like IL-1α, IL-9, IL-4, IL-6, granulocyte-macrophage colony-stimulating factor, and macrophage colony-stimulating factor. Apart from infection and certain autoimmune conditions, microglial cells also produce various cytotoxic and proinflammatory factors, like IL-6, TNF-α, and prostaglandin E (PGE) in response to radiation therapy (Cole et al. 2020). Activated microglia produce neurotoxic substances and various cytokines, which in turn leads to neurodegenerative and inflammatory effects in the brain (Barichello et al. 2019; Carson et al. 2006). Since the brain exhibits a limited amount of neuro-regenerative potential, the loss of the considerable number of neurons *via* immune-mediated toxicity is bound to have a significant detrimental impact (Colton 2009).

Glial cells also express a suite of transporters and receptors for the uptake of neurotransmitters, including glutamate and GABA. This prevents excitotoxicity due to the accumulation of presynaptic-synaptic glutamate. Once taken up by the glial cells, glutamine synthase converts glutamate to glutamine, which is then used as a precursor for both glutamate and GABA (Lago-baldaia et al., 2020). Hence, glutamine synthetase-synthesizing glial cells are an essential component of glutamatergic and GABAergic synapses (Bernstein et al. 2015), and activation of these cells will directly impact these synapses as well.

### Inflammatory markers and schizophrenia: clinical evidence

The cytokine hypothesis explains the positive relationship between pro-inflammatory markers and schizophrenia (Goldsmith et al. 2016). Cytokines arising due to both external and internal factors are known to be associated with various pathophysiological processes in the development and progression of schizophrenia, thus leading to a vast array of schizophrenic symptoms (Sommer et al. 2014). These symptoms surface during the phase of late adolescence and continue throughout life.

Shreds of evidence from preclinical and clinical studies have shown persistent elevation in various inflammatory markers like TNF-α, IL-12, IL-1β, IL-6, IL-1, and interferon (IFN)-γ in first onset and acute relapse schizophrenia subjects who were on antipsychotic medications (Miller et al. 2011). Inflammatory markers like cytokines are significant regulators of immune reactions and orchestrate brain development. A drastic rise in their level is seen in medication naïve psychosis patients (Juncal-Ruiz et al. 2018; Upthegrove et al. 2014) and the post-mortem brains of schizophrenic patients (Van Kesteren et al. 2017). These studies indicate a positive association between inflammation and the development of psychosis. A recent meta-analysis showed a positive relationship between inflammatory biomarkers and severity of negative symptoms in the antipsychotic naïve first episode of psychosis (FEP) population (Dunleavy et al. 2022). Meta-analysis has shown elevated levels of pro- and anti-inflammatory cytokines in CSF of relapsed as well as FEP patients, with IL-6 being in the highest concentration along with IL-1, IL-8, IL-1β, TNF-α and TGF-β (Upthegrove et al. 2014; Gallego et al. 2018). In addition to these cytokines, CSF kynurenic acid (KYNA) was elevated in major psychiatric disorders (Wang and Miller 2018). These markers were found to decline after treatment with antipsychotics. This suggests that anti-inflammatory pharmacological interventions may improve clinical outcomes in schizophrenia patients compared to treatment with antipsychotics alone on account of dose-related constraints and mixed effects of the latter. These inflammatory markers are expressed differently and provide unique contributions to the pathophysiological process of schizophrenia. IL-6 which is found to be highly expressed in psychotic patients (Delaney et al. 2019) is associated with increased oxidative stress and might have a possible association with GABAergic dysfunction in the schizophrenia brain (Behrens et al. 2008).

A study on 41 schizophrenic patients with acute psychosis revealed a significant elevation in the levels of IL-6, IL-2R, and IL-8 compared with the control. A positive correlation was found between symptom severity and levels of IL-6 and IL-2R (Dahan et al. 2018). Another case-control study conducted on at-risk mental state subjects (*n* = 17), patients with psychotic disorder (*n* = 77) and healthy controls (*n* = 25) tracked the transition of at-risk mental state subjects to psychosis for a period of 24 months. During this period, 6 out of 17 at-risk mental state subjects who developed psychosis showed an increase in IL-6 levels compared to those who did not. This explains the possibility of IL-6 being a biomarker for early psychotic symptoms; however, studies on large sample sizes are warranted to prove this (Stojanovic et al. 2014). Further, a positive correlation is associated with IL- 1 levels and duration of illness, with a significant rise in its levels during the initial stages of illness (Möller et al. 2015). IL-1β marker is known to initiate the conversion of progenitor cells into dopamine neurons (Potter et al. 1999). Hence a constructive correlation has been found between the severity of schizophrenia and various inflammatory markers (Liu et al. 2010).

Recent studies have suggested that antipsychotic drugs can modulate different immune reactions and Brain Derived Neurotrophic Factor (BDNF) based upon duration of treatment. For instance, 6-week treatment with olanzapine reduced proinflammatory cytokines such as IL-1 β, IFN-γ, IL-6 and TNF-α levels, in schizophrenia patients and increased the proinflammatory cytokines such as IL-10 (Zhao et al. 2024). Additionally, research indicated that eight weeks of atypical antipsychotics treatment significantly reduced blood pro- and anti-inflammatory cytokines in first-episode drug-free schizophrenia patients (Chen et al. 2021).

However, the relationship between antipsychotic treatment and BDNF levels remains to be understood further (Alvarez-Herrera et al. 2020). Zhao et al. 2023; observed treatment of first-episode schizophrenia patients with risperidone for a period of two weeks, led to a significant increase in the BDNF levels along with a decrease in psychotic symptoms (Zhao et al. 2023). In schizophrenic patients, 40 days treatment with risperidone exerted more efficacy than clozapine in decreasing of BDNF levels (Ajami et al., 2014). In contrast, olanzapine or risperidone did not show any significant change in BDNF during a 6-week prospective study in acute schizophrenia patients (Kudlek Mikulic et al. 2017).

A subset of pre-clinical studies belongs to immune activation during the maternal gestational period. These studies emphasized the correlation between maternal immune activation (MIA) during the gestational period and increased incidence of schizophrenia in the lifetime of the offspring. Any condition that activates an immune response in a pregnant mother and increases inflammatory markers is known to increase the risk of developing schizophrenia in the children born to the respective mother during MIA. Apart from this, there is evidence of alteration in neurotransmitter levels after an increase in the concentration of inflammatory cytokines following infection. Also, the development of psychosis and other psychiatric complications after the administration of vaccines and in people infected with human immunodeficiency virus (HIV-1) has been reported (Alciati et al. 2001). Notably, in a case study considering 16 males and 6 females infected with HIV, 8 (36.36%) developed psychotic symptoms. The CSF KYNA was found to be doubled in terms of concentration in patients who had psychotic symptoms compared to those individuals who did not have any psychotic symptoms (Atlas et al. 2007).

In the current context of COVID-19, a large number of individuals infected with SARS-CoV-2 virus have been found to present high rates of psychiatric disorders (Lim et al. 2020; Rentero et al. 2021; Xiao et al. 2022). These disorders include anxiety, mood disorders and symptoms characterized by structural delusional disbelief, thoughts of references, etc., similar to symptoms seen in patients with schizophrenia. A 78-year-old patient who had undergone a kidney transplant and was taking tacrolimus as an immunosuppressant suddenly developed psychotic symptoms. On admission to the hospital, she was found SARS- COV2 positive. Concentrations of IL- 6, IFN-γ -induced protein and IL-8 were found to be high in the CSF of this patient compared to the control samples. The patient was administered hydroxychloroquine for nine days, following which her condition initially improved and again worsened on the thirteenth day. Administration of IL-6 inhibitor, tocilizumab led to an overall improvement in her mental status (Farhadian et al. 2020). These manifestations of psychotic symptoms corroborated the influence of inflammatory markers, due to the damage caused by the virus since studies have shown an increase in inflammatory markers in patients infected with SARS- COV2 infection (Takahashi et al. 2020). Similarly, KYNA: KYA ratio was found to be higher in SARS- COV2 infected patients who have high inflammatory markers (Cai et al. 2021). These inflammatory markers must have altered the various pathways of schizophrenia, resulting in psychotic symptoms.

A few case reports have also been reported about patients developing psychotic symptoms after the administration of various vaccines, including the COVID-19 vaccine (Kuhlman et al. 2018; Reinfeld et al. 2021; Romeo et al. 2021). Although most of these reports did not mention the alterations in the cytokine levels, it is a possibility that the alteration in the cytokines after the administration of the vaccine might have led to the development of psychotic symptoms. Meta-analysis studies have proven the effectiveness of antipsychotics in reducing the levels of the inflammatory marker in schizophrenia subjects (Romeo et al. 2018). In a case-control study carried out on 38 patients with the FEP without any previous exposure to antipsychotic treatment, untreated patients with the FEP had a higher concentration of EGF (endothelial growth factors), IL-4, IL-6, IL gamma, and IFN-γ compared to healthy controls. With administration of antipsychotics, the EGF, IL-2 and IL-4 were significantly reduced along with commendable clinical improvement and suppression of schizophrenic symptoms (Haring et al. 2015). Therefore, the addition of an immunomodulatory adjuvant therapy might prove to be beneficial for psychosis patients and may be considered as an effective treatment strategy for better disease outcomes.

### Effect of inflammatory markers on various pathways of schizophrenia

#### Kynurenic acid pathway and Schizophrenia

**Fig. 1:**
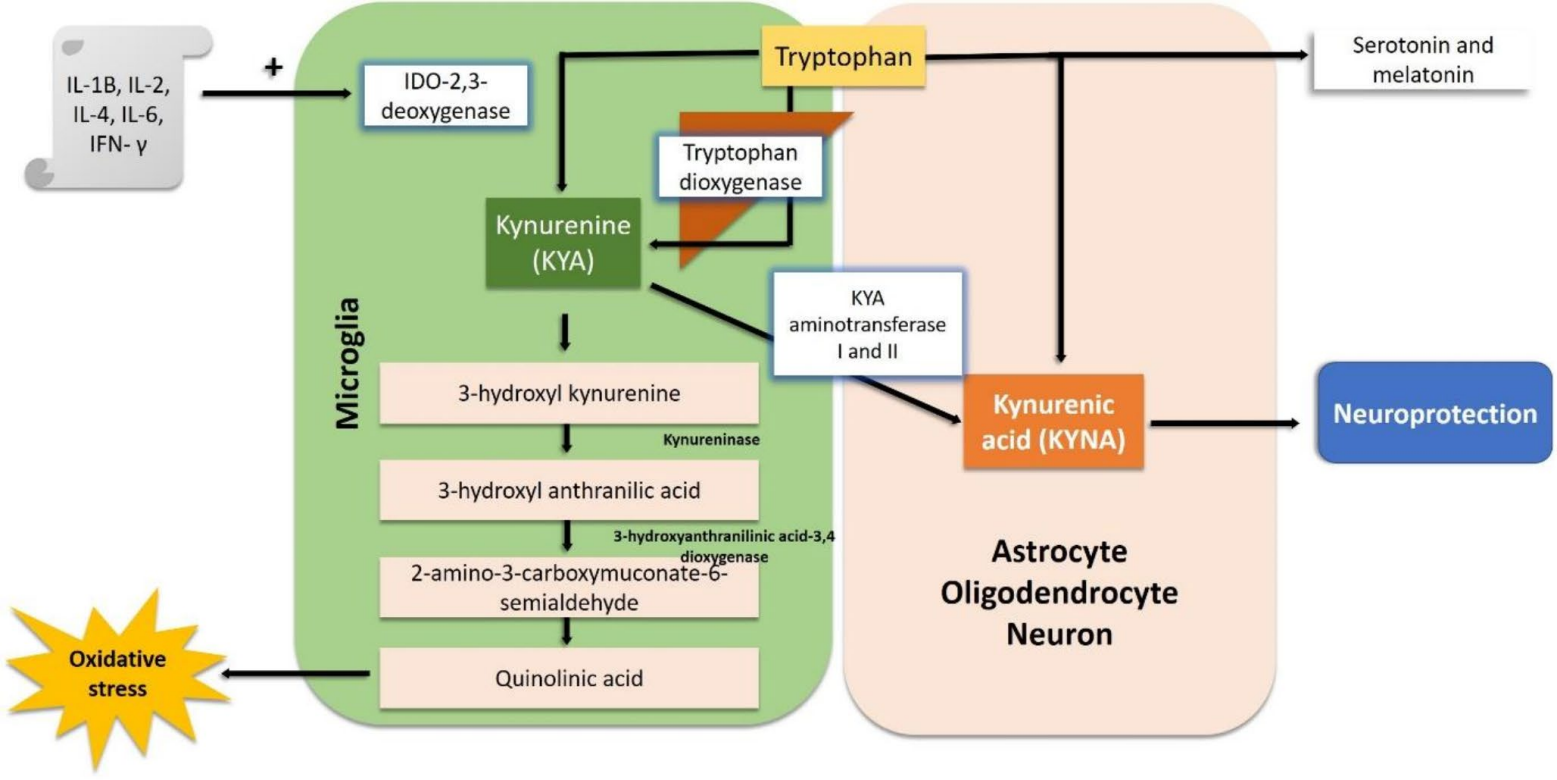
The kynurenic acid hypothesis of schizophrenia. Impaired kynurenine pathway metabolism occurs in the prefrontal cortex of individuals with schizophrenia

This hypothesis is based on KYNA being a broad-spectrum glutamate receptor antagonist that occurs naturally in the brain. An elevated concentration of this compound might initiate psychomimetic behavior in humans (Erhardt et al. 2007). In support of this hypothesis, various studies have shown the elevated concentration of KYNA in schizophrenic subjects (Erhardt et al. 2007; Plitman et al. 2017). Around 60% of KYNA is produced in the peripheral region and then transferred into the brain *via* amino acid transporters present in the BBB. The other 40% is produced inside the brain by activated glial and immune cells (Vécsei et al. 2013). CSF KYA and KYNA have been found in multiple folds in schizophrenic brains. A case-control study considered 16 verified schizophrenic males in the age group 23–49 years and 29 healthy males in the age group of 18–59 years. The CSF was withdrawn by lumbar puncture after 8 h of fasting and was centrifuged before analyzing for the levels of KYNA and KYA. The results showed elevated levels of CSF KYNA and almost double concentration of CSF KYA in schizophrenic males (Linderholm et al. 2012). KYNA is the degradation product of tryptophan, contributing about 90% towards the degradation product of tryptophan metabolism (Plitman et al., 2017). This pathway is highly sensitive to inflammatory mediators (Schwarcz et al. 2012). About 5% of the tryptophan gets converted into the neurotransmitter serotonin and the hormone melatonin, while the remaining 95% gets converted into N- formyl kynurenine with the help of tryptophan dioxygenase in the liver and indoleamine 2,3-dioxygenase (IDO) in the immune system and brain. The N-formyl kynurenine generated is then converted into KYA with the help of the enzyme formamidase (Lawson et al. 2013; Muneer 2020). KYA in turn gets converted into 3-hydroxy kynurenine and subsequently into 3- hydroxyanthranillic acid with the help of the enzymes kynurenine hydroxy mono-oxygenase and kynureninase, respectively. This 3-hydroxyanthranilic acid with the help of enzyme 3- hydroxyanthranilate-3,4-dioxygenase, gets converted into 2-amino-3-carboxymuconic-6-semialdehyde which then undergoes non-enzymatic cyclization to produce quinolinic acid, which is further converted into nicotinamide adenine dinucleotide (NAD+). 2-amino-3- carboxymuconic-6-semialdehyde can also undergo nonenzymic cyclization to produce acetyl CoA (Badawy 2017). 3- hydroxykynurenine, along with its downstream catabolites and quinolinic acid, are produced in the microglia, whereas KYNA synthesis is carried out in astrocytes, oligodendrocytes and neurons (Tutakhail et al. 2020). The rate-limiting step in this pathway is the conversion of tryptophan to KYA, which is catalyzed by the enzymes IDO and tryptophan dioxygenase (Wu et al. 2014). Elevated KYA and KYNA in schizophrenic patients are due to the induction of IDO and tryptophan 2,3 dioxygenase by IFN-γ and various other inflammatory markers such as TNF-α, IL-6 (Chen 2011) (Fig. 1). A 6-week double-blind placebo-controlled randomized clinical trial considering 100 inpatients with first-episode drug-naive schizophrenia, all of whose IDO levels and the levels of six other inflammatory cytokines IL-1β, IL-6, TNF-α IL-17, IL-4, and IFN-γ were higher than the healthy subjects, all individuals in this study group were administered celecoxib (a COX- 2 inhibitor) or placebo combined with risperidone. Over the six weeks treatment period a significant reduction in IDO and IL-1β, IL-6, TNF-α IL-17, IL-4, IFN-γ levels and a corresponding improvement in positive and negative symptoms were found in the celecoxib-treated group compared to the placebo group (Zhang et al. 2021).

The elevated metabolites of the KYA pathway are associated with various neuropsychiatric disorders. They lead to N-methyl-D-aspartate (NMDA) receptor hypofunction resulting in psychotic symptoms (Erhardt et al. 2001). Elevation of KYNA is also associated with an increased firing from the dopaminergic neurons of the midbrain mediated through inhibition of GABAergic input, which would otherwise dampen the dopamine neuron activity in the midbrain (Erhardt et al. 2002). Quinolinic acid produced in this pathway is an NMDA receptor agonist (Walker et al. 2013; (Johansson et al. 2013). As a result, it increases the glutamate concentration in the synaptic region by reducing its reuptake by astrocytes, which results in excitotoxic effects in the CNS (Laugeray et al. 2010).

#### Dopamine, inflammation, and schizophrenia

The classical dopamine hypothesis states that hyperactive dopaminergic transmission in various brain parts due to increased density and sensitivity of dopamine 2 (D2) receptors is responsible for the development of schizophrenia. Contrary to this, the modern dopamine hypothesis states that low dopamine levels in the medial prefrontal cortex and increased dopamine levels in the mesolimbic pathway are responsible for negative and positive symptoms of schizophrenia respectively. The unifying hypothesis of dopamine proposes a common pathway that states that multiple risk factors like genetic, environmental, stress and various others interact and cause dopamine dysregulation in the striatal region, altering signal transmission and cause psychosis (Davis et al. 1991; Howes et al. 2012).

A clinical study concluded that innate immune activation and the release of inflammatory cytokine IL-6 following typhoid vaccination affects reward circuits and substantia nigra function, thus showing significant psychomotor consequences (Brydon et al. 2008). Similarly, studies have also concluded that change in behavior following peripheral administration of IFN-α and various other cytokines is mediated via altered dopaminergic neurotransmission (Shuto et al. 1997; Song et al. 1999). There are various dopamine pathways present in the brain, and alteration of dopamine levels in these pathways are associated with various schizophrenic symptoms. The nigrostriatal pathway that extends from substantia nigra to the caudate nucleus, hypo-activity of this pathway results in extrapyramidal symptoms, hypo-activity or under activity of mesocortical pathway which extends from ventral tegmental area to cortex is known to cause negative, cognitive and affective symptoms of schizophrenia. On the other hand, hyperactivity of mesolimbic pathway is known to mediate positive symptoms of Schizophrenia (Brisch et al. 2014; Patel et al. 2014; Stahl 2007).

Dopamine levels are managed by the following transporters: the membranous dopamine transporter (DAT) and the vesicular monoamine transporter 2 (VMAT2) (Iliopoulou et al. 2021). Dopamine primarily produces its effect by activating G protein-coupled dopamine receptors, which are classified into two groups, i.e., the D1 subgroup containing D1 and D5 and the D2 subgroup containing D2, D3, and D4 (Beaulieu and Gainetdinov 2011) each of these subgroups having a different affinity for dopamine (Mittal et al. 2017). Phenylalanine acts as a precursor for dopamine synthesis. Phenylalanine is converted to tyrosine by phenylalanine hydroxylase in the presence of tetrahydrobiopterin (BH4) as a cofactor. Tyrosine is converted into L- dopa in the presence of tyrosine hydroxylase (TH). Again, in the presence of BH4 as a cofactor, L- dopa gets converted into dopamine in the presence of dopamine decarboxylase (Cunnington and Channon 2010). The cofactor BH4 is a pteridine derivative and is produced by the action of the enzyme guanosine triphosphate (GTP)-cyclo-hydrolase-1. In human monocyte-derived macrophages and dendritic cells, this enzyme, which is highly susceptible to inflammatory markers, produces neopterin instead of BH4, in the presence of inflammatory markers (Geisler et al. 2013), resulting in a significant reduction of BH4 levels (Richardson et al. 2005, 2007) which in turn leads to a reduction in the synthesis of dopamine (Geisler et al. 2013). Peripheral monocytes, T- cells and microglia express dopaminergic receptors. High levels of dopamine stimulate low affinity dopamine receptors including D1, D2, and D4 to induce anti-inflammatory effects on immune cells. On the other hand, reduced dopamine levels stimulate D3 and D5 receptors which are high affinity dopamine receptors and hence trigger an inflammatory response. This effect of altered dopamine levels on the immune system leads to stress and other behavioural changes (Vidal and Pacheco 2020). Apart from expressing dopaminergic receptors on their surface T- cells also have TH, DAT, VMAT2, and catechol-O-methyltransferase, suggesting they have the capacity to uptake, synthesize, store, and release dopamine (Bergquist et al. 1994; Cosentino et al. 2007; Qiu et al. 2004). A study carried on mRNA of human monocyte derived macrophages, indicated the presence of all dopamine receptor subtypes along with DAT, VMAT2, TH and aromatic amino acid decarboxylase (AADC) (Gaskill et al. 2012). Some antipsychotics exert their pharmacological action by blocking the synthesis or storage of dopamine. Antipsychotics like reserpine act by inhibiting VMAT-2, thus preventing the dopamine from getting stored in the storage vesicles and letting it get depleted in the cytoplasm. Administration of the TH inhibitor, alpha methyl para tyrosine, inhibited the synthesis of dopamine, thus reducing the symptoms of schizophrenia (Uno and Coyle 2019). An in vivo study using four rhesus monkeys has shown enhanced striatal extra-synaptic dopamine levels in schizophrenic condition using photon emission tomography studies (Breier et al. 1997). Although no association is found between dopamine active transporters density in the etiology or treatment of schizophrenia (Fusar-Poli and Meyer-Lindenberg 2013), PET neuron-imaging study carried using 18 F-DOPA showed enhanced dopamine production in the dorsal striatum in individuals with ultra-high risk of psychosis (Egerton et al. 2013).

Inflammatory markers like IFN-γ are known to play a significant role in altering dopamine availability and binding; nonhuman primates four weeks after the administration of these inflammatory markers have shown reduced dopamine release from the striatum along with decreased dopamine binding to D2 receptors (Felger et al. 2013). Patients who were suffering from HCV infection when administered with IFN and ribavarin, showed a significant reduction in the activity of the ventral striatum and reduced dopamine turnover in the caudate and putamen. This was accompanied by a significant increase in bilateral radio labeled fluorodopa (^18^F-DOPA) uptake in the caudate and putamen (Capuron et al. 2012). In most human cells (Geisler et al. 2013), IFN-γ induces the production of GTP cyclo-hydrolase-I, which initiates the conversion of GTP required for the biosynthesis of 5,6,7,8- BH4. It is a cofactor of many enzymes like phenylalanine-4-hydroxy lase, tyrosine-5-hydroxylase, and nitric oxide synthases. These enzymes play an essential role in the synthesis of neurotransmitters. Thus, activation of GTP cyclohydrolase-I by IFN-γ indirectly leads to enhanced dopamine production by increasing the synthesis of BH4 (Neurauter et al. 2008). On the other hand, activation of GTP- cyclo-hydrolase- by IFN-γ in the human macrophages/ monocytes, which are relatively deficient of 6- pyruvoyl tetrahydrobiopterin synthase, leads to the accumulation of 7,8- dihydro-neopterin triphosphate which subsequently gets converted into neopterin and 7,8 dihydro-neopterin in the presence of phosphates. Thus, neopterin is produced at the expense of tetrahydropteridine (BH4) (Murr et al. 2002; Wirleitner et al. 2002) which is known to produce reactive oxygen species (Razumovitch et al. 2003). These reactive oxygen species readily oxidize oxidation labile BH4 hence resulting in reduced dopamine production (Neurauter et al. 2008).

#### Inflammation, glutamate and schizophrenia

The glutamate theory of schizophrenia was developed after observing the ability NMDA receptor antagonists to induce psychiatric symptoms. This theory states that hyper-glutamatergic activity in the synaptic regions is responsible for the development of negative, positive, and cognitive symptoms of schizophrenia (Moghaddam & Javitt 2012). Drugs that non-competitively antagonize the NMDA receptors produce positive, negative and cognitive symptoms in healthy individuals, suggesting a close association between dysregulated glutamate receptors and schizophrenia (Paoletti et al. 2013).

**Fig. 2:**
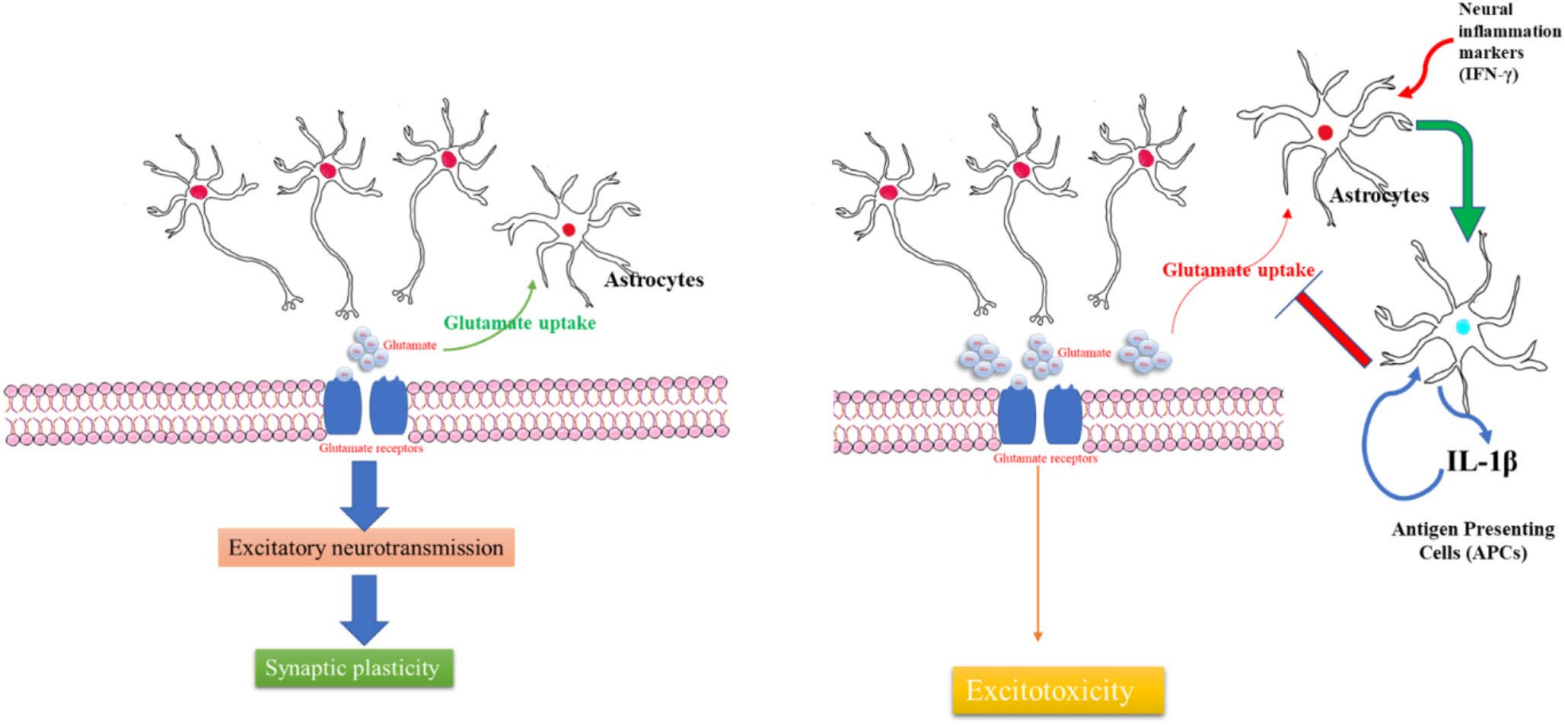
Impact of neural inflammation on glutamate excitotoxicity

This theory is further strengthened by various preclinical trials conducted on rats where systemic administration of NMDA receptor antagonists to rats at doses sufficient to impair cognitive functions and motor stereotypy led to increased glutamate efflux in various regions of the brain (Lorrain et al. 2003). This is similar to that observed in schizophrenia patients, using magnetic resonance spectroscopy (Merritt et al. 2021). Glutamate is an excitatory neurotransmitter acting on the brain and spinal cord *via* ionotropic glutamate receptors (Paoletti et al. 2013). It performs various activities like controlling the maturation and formation of the synapse, acts as a substrate for the source of energy in oxidative metabolism, and guides various processes in the developing brain (Ryan et al. 2021). Glutamate is produced in pre-synaptic-synaptic neurons, and then vesicular glutamate transporters (VGluT) pack them into secretary vesicles; when the pre-synaptic neurons get excited, they fuse with secretary vesicles and release glutamate into the synaptic cleft. The released glutamate acts on various glutamate receptors and causes rapid clearance of extracellular glutamate, which then either enters the TCA cycle or forms glutamine (Rubio et al. 2012). These receptors are classified into α-amino 3-hydroxy-5-methyl-4-isoxazole propionic acid (AMPA), kainite receptors, and NMDA receptors (Nakanishi 1992). NMDA receptors are glutamate-gated and have three different subunits Glu1, Glu2, and Glu3. Any malfunction in these NMDA receptors results in various psychological and neurological illnesses. NMDA receptors are glutamate-gated heterotetrameric receptor complexes composed of Glu1, Glu2, and Glu3 subunits and mediate glutamatergic excitatory neurotransmission, hence inducing synaptic plasticity (Paoletti et al. 2013). These receptors are activated by simultaneous binding of glutamate and glycine along with depolarization of the membrane potential, to relieve voltage-dependent magnesium block (Zhu et al. 2016). The glutamate released during the action potential is produced from glutamine supplied by astrocytes (Coyle 2006). Astrocytes in the CNS are subdivided into three types, viz., radial, protoplasmic and fibrous astrocytes (Liddelow and Barres 2015). Among these, protoplasmic astrocytes constitute branching structures, which are further subdivided into finer processes. These processes present on one side of the protoplasmic astrocytes, encapsulate several thousands of synapses, thus producing astrocytic cradles, and the astrocytic cradles that surround the synaptic region have a high distribution of glutamate transporters in them, which play a pivotal role in glutamate clearance (Bushong et al. 2002; Sofroniew and Vinters 2010). Once the glutamate transporters take up the glutamate, it is converted into the inert intermediate glutamine. Glutamine once released, is absorbed by neurons and gets converted into glutamate, which inside the neurons is packed inside the synaptic storage vesicles by the vesicular transporter (VGlu-3). This synaptically-stored glutamate acts as the primary source of glutamate during neurotransmission (Danbolt 2001). Apart from expressing glutamate receptors, astrocytes also express receptors sensitive to various inflammatory mediators like cytokines and chemokines (Hsuchou et al. 2009; Pan et al. 2012). During diseased state, increased inflammatory cytokines like IFN-γ transform these astrocytes into APCs and phagocytic cells, delineated by the elevation of markers of inflammatory activation (toll-like receptors (TLRs), MHC class II receptors, and an intracellular immune receptor system containing the Rig-1-protein complex of the inflammasomes). These inflammasomes are intracellular complexes that can sense danger-associated molecular patterns, which once activated result in the activation of caspase-1, thus converting pro-IL-1 into IL-1β. IL-1β in-turn can activate astrocytes, thus making the astrocytes release a large number of inflammatory mediators, and this state of astrocytes is associated with oxidative stress as well as reduced glutamate clearance, which contributes to excitotoxicity. Astrocytes are divided into two subtypes, viz., A1 and A2. Of these, the A1 type can be induced by inflammatory stimuli (Zamanian et al. 2012). These A1 subtypes are further pathologically subclassified into two categories viz., loss of function type, in which the astrocytes stop performing their normal function (glutamate clearance and/or synaptogenesis), and the gain of function type, wherein the astrocytes exhibit deleterious activities like synaptic destruction, which normally does not occur in resting astrocytes (Sofroniew and Vinters 2010). Once the inflammatory mediators induce these A1 astrocytes, they exhibit their neurotoxic effect by upregulating the components of the complementary cascade, thus causing glutamate-mediated excitotoxicity and inducing oxidative stress (Zamanian et al. 2012) (Fig. 2).

### Gut-brain axis and Schizophrenia

As reported earlier, schizophrenia has been associated with infections contributing to gut dysbiosis (Ju et al. 2023). There exists a complex communication network between the gut and the CNS, which is known as the gut-brain axis. An altered function of this axis, also known as gut dysbiosis, leads to increased permeability of the gut and a rise in systemic inflammation, exacerbating peripheral and central neuroinflammation. Reduction in the gut microbiota diversity in schizophrenic patients, was supported further with preclinical evidence of reduced glutamate and increased GABA (Zheng et al. 2019). Thus, gut dysbiosis predisposes altered glutamate and GABA neurotransmission including tryptophan and serotonin pathway with superimposed neuroinflammation. These factors trigger dopaminergic overactivity in the brain, for manifestating psychosis (Theleritis et al. 2024).

Multiple factors can initiate dysbiosis, including environmental pollutants, antibiotic use and infections that compromise the integrity of the gut, alter the immune responses and result in systemic inflammation (Kearns 2024). Elevated levels of lipopolysaccharides (LPS) and immunoglobulins observed in various neurological conditions support the hypothesis that alteration in gastrointestinal permeability might trigger systemic inflammatory responses (Ghosh et al. 2020). In the brain, the kynurenine pathway, which serves as the primary route for tryptophan metabolism, is significantly impacted by this process. This dysregulation of the kynurenine pathway due to inflammation leads to the production of quinolinic acid- a neurotoxic metabolite; this Quinolinic acid produced in this pathway is an NMDA receptor agonist (Walker et al. 2013; Johansson et al. 2013). As a result, it increases the glutamate concentration in the synaptic region by reducing its reuptake by astrocytes, which results in excitotoxic effects in the CNS (Laugeray et al. 2010).

The future research may envisage newer therapeutic approaches based upon reversing gut dysbiosis and associated neurotransmitter imbalance to curb schizophrenia effectively. One of the promising approach is using psychobiotics, which may confer efficacy by administering commensal gut bacteria as probiotics (Sarkar et al. 2016). Psychobiotics can regulate neurotransmitters by improving diversity for gut microbiota and thereby correcting neurotransmitters imbalances and neuroinflammation. Administration of probiotics and prebiotics, that help in maintaining a healthy gut microbiota, has shown promising results in reducing gut permeability as well as systemic inflammation, and this could be used as an adjuvant therapy to offer a protective effect to the brain by preventing the production of quinolinic acid (Dinan et al. 2013; Sarkar et al. 2016; Xiang et al. 2022).

### Scope of anti-inflammatory drugs in the treatment of schizophrenia

Pharmacotherapy with antipsychotics is the mainstay of therapy for schizophrenia. These agents are commonly classified into first-generation (or typical) and second-generation (or atypical) antipsychotics. The former are dopamine receptor antagonists, and the latter are serotonin-dopamine antagonists. The older first-generation agents are associated with extrapyramidal motor side effects and elevation of prolactin levels (Huhn et al. 2019). In the past, immune system modulators have shown promising results as adjuvant therapy, especially when administered during the initial phase of illness. N-acetyl cysteine (NAC) is the most commonly used immunomodulatory agent for this purpose (Pardo-de-Santayana et al. 2021). Many studies have also pointed out the possible anti-inflammatory effect of antipsychotic medications, indicating their role as immunomodulatory agents, along with their action on the dopaminergic and serotonergic systems (Table 1). However, many of these agents also demonstrate induction of pro-inflammatory cytokines and factors, indicating a mixed effect.

**Table 1:** Effect of currently used antipsychotics on inflammatory marker(s) and possible significance on schizophrenic effects

Antipsychotic	Inflammatory marker(s) affected	Model	Observations/ Significance	Reference
1st generation antipsychotics				
Haloperidol	TNF-α, IL-8, IL-6	Human dendritic cells (DC)	Dose dependent decrease in the production of TNF-α observed in mature DC with haloperidol No significant effect on IL-8, IL-6 production in mature/ immature DC	Chen et al. 2012
	IL-4, IL-10, IFN-γ	Lipopolysaccharide (LPS)-activated peripheral blood mononuclear cells (PBMC) blood culture from FEP patients	Increase in anti-inflammatory markers: IL-4 and IL-10 Decrease in pro-inflammatory marker IFN-γ	Al-Amin et al. 2013
	Inducible nitric oxide synthase (iNOS) (pro-inflammatory)	Murine BV-2 microglial cell lines	Decrease in production of iNOS However, no significant effect seen on levels of anti-inflammatory markers	Racki et al. 2021
	CRP	Human subjects	Increase in median CRP levels after 3 months of treatment with haloperidol compared to baseline levels; however, no net change after 12 months	Diaz et al. 2010
Chlorpromazine	IL-1β and IL-2	Mixed glial cell and microglial cell cultures of rat	Reduction in IL-1β release after 1 and 3 days of exposure at 2 and 20 µM doses, respectively Reduction in IL-1 secretion after 3 days of incubation at 0.2, 2 and 20 µM doses Results of the study suggest that chlorpromazine administration may influence microglial activity in the brain of schizophrenic human subjects	Labuzek et al. 2005
Flupentixol	IL-1β and IL-2	Mixed glial and microglial cell cultures of rat	Reduction in concentration of IL-1β and IL-2 after 1 or 3 days of flupentixol exposure at neuroleptic doses (0.2, 2 and 20 µM)	Kowalski et al. 2004
Trifluperidol	IL-1β and IL-2	Mixed glial and microglial cell cultures of rat	Reduction in concentration of IL-1β and IL-2 after 1 or 3 days of trifluperidol exposure at neuroleptic doses (0.2, 2 and 20 µM)	Kowalski et al. 2004
Spiperone	TNF-α, iNOS and IL- 1β	Murine BV-2 microglial cell lines	Attenuation in the expression of iNOs, IL- 1β and TNF-α genes Attenuation in lipopolysaccharide (LPS)-induced nuclear factor kappa-B (NF-kB) and p38 mitogen-activated protein kinases (p38 MAPK) (which are inducers of pro-inflammatory cytokines and iNOS in glial cells) Attenuation in NO production in ATP-stimulated primary microglial cultures Reduction in neuroblastoma cell death by activated microglia (neuroprotective effect)	Zheng et al. 2008
2nd generation antipsychotics				
Olanzapine	CRP	Human subjects	No significant difference between median CRP levels at base line and after both 3 months and 12 months	Diaz et al. 2010
	NO	LPS-activated murine N9 microglial cell line	Suppression of LPS-induced microglial cell activation and NO release Study proposes this to be a mechanism for therapeutic action of olanzapine in schizophrenia	Hou et al. 2006
Clozapine	Total COX activity, PGE_2_, calcium-independent phospholipase A2 (iPLA_2_), brain derived neurotrophic factor (BDNF) and drebrin	Male CDF-344 rats	Reduction in COX-1 and total COX activities in brain Reduction in concentration of pro-inflammatory PGE-_2_ in brain Increase in anti-inflammatory BDNF expression levels in brain Increased production of anti-inflammatory docosahexaenoic acid (DHA) metabolites in brain (evidenced from iPLA_2_ expression) Increase in expression of postsynaptic marker, drebrin (neuroprotective)	Kim et al. 2012
	iNOS	Neonatal male Wistar rats	Reduction in iNOS immunoexpression in striatal and hippocampus areas Reversal of alteration caused due to poly(I: C) induced microglial activation	Ribeiro et al. 2013
	IL-4, IL- 10 and IFN-γ	LPS-induced peripheral blood mononuclear cells blood culture from FEP patients	Increase in anti-inflammatory markers: IL-4 and IL-10 Decrease in pro-inflammatory marker IFN-γ	Al-Amin et al. 2013
Risperidone	TNF-α, IL- 6, IL- 10 and IL- 4	Human subjects (FEP)	Normalization of elevated IL-6, IL-10 and TNF-α to the levels of those in healthy controls However, significant reduction in IL-4 compared to healthy controls after 10 weeks of treatment	Noto et al. 2014
	IFN-γ-inducible protein 10 (IP-10) and IL-12	Human dendritic cells (in vitro)	Dose dependent decrease in the production of inflammatory mediators IL-12 and IP-10 Decrease in the production of TNF-α at low dose (10^−8^) M and increase in production TNF-α at high dose (10^−6^–10^−5^) M of risperidone Dose dependent increase in the production of 1 L-10, IL-8 and IL- 6	Chen et al. 2012
	CRP	Human subjects	Significant reduction in median CRP levels compared to median baseline CRP levels after 3 months of treatment with risperidone	Diaz et al. 2010
	IL-1β, iNOS, COX, TNF-α	LPS-administered male Wistar Hannover rats	Prevention of increased inflammatory parameters viz., IL-1β, TNF-α, activity of iNOS, COX, p38 MAPK and NF-kB in brain cortex Restoration of levels of deoxyprostaglandins and peroxisome proliferator activated receptor γ	MacDowell et al. 2013
	iNOS	Murine BV-2 microglial cell line	Reduction in production of iNOS Increase in mTORC1 activity compared to control (evidenced by ratio of phosphorylated p70S6K (p-p70S6K) to p70S6K) > > Could be pro-inflammatory	Racki et al. 2021.
Aripiprazole	NO, TNF-α	Murine microglial cell line 6 − 3	Reduction in NO and TNF-α in both IFN-γ-activated and LPS-activated microglia, possibly *via* suppression of intracellular Ca2^+^ (required for release of NO and some cytokines)	Kato et al. 2008
	iNOS, CD86 (pro-inflammatory phenotype cell-surface markers), CD206 (anti-inflammatory phenotype marker)	Murine BV-2 microglial cell line	Reduction in production of iNOS Increase in expression of CD206 and CD86 Reduction in mTORC1 activity compared to control (evidenced by ratio of phosphorylated p70S6K (p-p70S6K) to p70S6K) > > Lesser inflammatory action	Racki et al. 2021.
	CRP, IL-1β, IL-6, TNF-α, sTNF-R1, IL-12, IL-23, IL-1Ra, TGF-β1, IL-4, IFN-γ, IL- 10	Human subjects	Reduction in pro-inflammatory markers IL-1β, IL-6, TNF-α, sTNF-R1, IL-12, IL-23*, IL-1Ra, IFN-γ and ratio of IFN-γ:IL-10, after 28 days of treatment Reduction in anti-inflammatory IL-4 and TGF-β1 Significant increase in anti- inflammatory marker IL-10 and / or reduction in ratio of IL-6:IL-10 A significant negative correlation observed between levels of IL-10 levels and PANSS (positive, negative and total) scores after the study *First study to investigate IL-23 levels (main cytokine in brain autoimmune inflammation)	Sobiś et al. 2015
Loxapine	IL-1β and IL-2	LPS-activated mixed glial cells of rat	Reduction in release IL-1β (at 0.2, 2, 10 and 20 µM doses) and IL-2 (at 2, 10 and 20 µM doses)	Labuzek et al. 2005
Quetiapine	IFN-γ, IL- 4 and IL- 10	LPS-activated peripheral blood mononuclear cells blood culture from FEP patients	Increase in anti-inflammatory markers: IL-4 and IL-10 Reduction in pro-inflammatory marker IFN-γ	Al-Amin et al. 2013
	CRP, IL-6, IL-10	Human subjects (with bipolar disorder)	Significant reduction in IL-16 and CRP and restoration to normal levels after 6 weeks of treatment Reduction in IL-10 and IFN-γ levels (not statistically significant)	Ferrari et al. 2022

Non-steroidal anti-inflammatory drugs (NSAIDs) have demonstrated beneficial effect in schizophrenia patients (Sommer et al. 2011). These agents mediate their actions by inhibiting the enzyme cyclo-oxygenase (COX) iso-enzyme, which is subdivided into COX-1 and COX-2. Unlike COX-1, COX-2 is not constitutively expressed in the body and is expressed only during an inflammatory response. Most NSAIDs are non-selective and act on both COX-1 and COX-2, except a few like celecoxib which are very specific to COX-2 and inhibits only COX-2 (Ghlichloo and Gerriets, 2023). A randomized, double-blind placebo control trial on 70 schizophrenic patients who were administered aspirin as an adjuvant therapy showed significant improvement in the symptoms of schizophrenia, especially with more remarkable improvement seen in those with higher alteration in the immune markers (Laan et al. 2010). A meta-analysis considering 62 randomized, double-blind trials which had 2914 patients meeting the inclusion criteria showed the significant role of aspirin in improving the overall positive and negative symptom score in the positive and negative syndrome scale when compared to antipsychotic drug monotherapy (Cho et al. 2019). Celecoxib has also shown significant improvement in positive, negative and general psychopathology scores even in first-episode schizophrenia patients. In a prospective randomized double-blind study conducted on 50 patients with acute exacerbation of schizophrenia, and who were randomly assigned into risperidone plus placebo and risperidone plus celecoxib group, patients who were in the risperidone plus celecoxib group showed a more significant improvement in symptoms in terms of positive and negative syndrome scale (Müller et al. 2002). There is contradicting evidence on the use of NSAIDs for improvement in schizophrenia. One meta-analysis study states that anti-inflammatory drugs lead to a significant improvement in positive and negative syndrome scale as well as sub-scores of the positive and negative symptoms of schizophrenia (Sommer et al. 2011). Conversely, another meta-analysis denied any such benefit of anti-inflammatory drugs over placebo on the positive and negative syndrome scale, when used along with antipsychotics (Nitta et al. 2013). Again, several randomized controlled trials have shown the benefit of aspirin in improving the negative symptoms, total positive and negative syndrome scale and general psychopathology in schizophrenia subjects (Laan et al. 2010). In a randomized double-blind placebo-controlled study conducted on 1000 schizophrenia patients, with antipsychotics and adjuvant therapy with either placebo or aspirin, the aspirin treated group had better clinical improvement in terms positive and negative syndrome scale, compared to placebo, after a follow-up period of three months. This effect was more prominent in patients with altered immune status (Laan et al. 2010). However, owing to the gastrointestinal and various other side effects anti-inflammatory drugs on long term use, a more detailed study is warranted with respect to the appropriate dosing of anti-inflammatory drugs in schizophrenia patients. Moreover, since the studies used to assess the benefit of anti-inflammatory drugs in schizophrenia were mostly done on a small sample size of patients and for a short period of time, more detailed studies considering large sample size and large duration should be considered to establish appropriate conclusions.

As discussed in previous sections, microglial activation plays a significant role in the inflammation and progression of schizophrenia. Minocycline, a second-generation tetracycline antibiotic with strong anti-inflammatory properties that inhibit microglial activation, can play a massive role in the management of schizophrenia. This is demonstrated in methamphetamine-induced psychotic mice models, wherein minocycline administration showed improvement in cognitive symptoms (Mizoguchi et al. 2008). Similarly, even in humans, minocycline has shown improvement in the negative symptoms of schizophrenia (Chen et al. 2018). Other meta-analysis studies have also proven the superiority of minocycline in resolving negative symptoms when compared to antipsychotic monotherapy alone (Cho et al. 2019; Solmi et al. 2017).

Apart from anti-inflammatory drugs, various other pharmacotherapeutic options that help to curb cytokine levels can be used as therapy adjuvants for schizophrenia. Antibody immunotherapy is currently used in various immune-related disorders like inflammatory bowel disease and rheumatoid arthritis, where the monoclonal antibodies (mAbs) target the cytokines and their receptors. (Chan and Carter 2010). In an 8-week open label clinical trial with the concomitant administration of i.v. tocilizumab (an IL-6 inhibitor) every 4 weeks along with the non-clozapine antipsychotic drugs, there was a significant improvement in BACS (brief assessment of cognition in schizophrenia), and verbal fluency after 4 weeks, compared to baseline levels. However, there was no improvement in the psychopathology, highly sensitive CRP or cytokine levels (Miller et al. 2016). In a randomized double-blind placebo-controlled trials considering 36 patients administered with 8 mg/kg of three-monthly doses of placebo or tocilizumab, there was no significant improvement in the positive negative or cognitive symptoms in the group which received tocilizumab when compared to the group that received the placebo. The authors state that this might be due to the inability of tocilizumab to cross the BBB (Girgis et al. 2018). This significant limitation of mAbs in having limited ability to cross the BBB is on account of their polarity and large size. On the contrary, in another randomized double-blind placebo-controlled study (Weickert et al. 2019) twenty seven schizophrenic patients with elevated peripheral inflammatory markers, were administered with subcutaneous injection of canakinumab 150 mg or placebo respectively. Here, canakinumab administration showed significant improvement in the positive symptoms when the trial participants were assessed in the eighth week. Most of these studies have been conducted on relatively small sample sizes. Due to the differences in the outcome of these different studies, it is necessary to conduct a study with large sample size and for a longer duration of action.

NAC is a glutathione precursor with both anti-inflammatory and antioxidant properties making it useful in the management of neurodegenerative diseases (Tardiolo et al. 2018). Abnormal amount of dopamine and glutamate metabolism in schizophrenic patients leads to oxidative stress resulting due to glutathione deficiency, which contributes to pathogenesis of schizophrenia (Tharoor et al. 2018). NAC exhibits both anti- inflammatory and neuroprotective function and has shown to stop the effect of inflammatory stimuli in rats with reduction in TNFα, IL-1β and IL-6 levels, (Palacio et al. 2011). Clinical studies have shown improvement in the Positive and Negative Syndrome Scale (PANSS) total and negative symptoms but no improvement in the PANSS positive score following the administration of NAC (Farokhnia et al. 2013; Tharoor et al. 2018). In a meta-analysis of randomized controlled trials of FEP and schizophrenia, NAC as adjuvant therapy for a period of 24 weeks or more, showed an improvement in the negative and total scores on the PANSS scale, along with some beneficial effect on the cognition in terms of working memory (Yolland et al. 2020). Besides this, studies have also shown the effect of drugs like statins, which possess anti-inflammatory properties, to improve the positive and negative symptoms of schizophrenia (Nomura et al. 2018). However, some other randomized controlled trials stated that there was no significant difference between statin and placebo groups (Ghanizadeh et al. 2014). Along with the improvement of schizophrenic symptoms, the lipid-lowering effect of statins might also help to reduce the cardiovascular side effects of antipsychotic drugs. Therefore, further detailed studies would be helpful in confirming the effectiveness of statins in schizophrenia patients. Apart from this, augmentation of estrogen therapy is also discussed as a possible approach in the management of schizophrenia, owing to reduction in disease severity seen in women who hit puberty and beyond, followed by increase in severity of the disease the women experience at or after menopause (Brand et al. 2021). However, more studies are needed to be done to decide on the implication of this therapeutic plan.

## Conclusion

There is a well-established relationship between inflammation and schizophrenia, at least in a subset of patients. If inflammation contributes even partly to the pathophysiology of schizophrenia, then treatments as ordinary as conventional anti-inflammatory drugs may strike at the root of the disease process. Inflammatory hypotheses converge with the existing hypothesis such as the dopaminergic and glutamatergic origin. The addition of anti-inflammatory drugs might enable clinicians to reduce the dose of antipsychotic drugs which will further help in reducing the prevalence of treatment-resistant schizophrenia. Since antipsychotics cannot be combined for their increased effectiveness due to their similar mechanism of action and side effects, the addition of other immunomodulatory agents along with the antipsychotics might prove as a promising treatment option. Notwithstanding, further studies conducted in-depth are required to find an effective immunomodulatory drug targeting the designated pathway associated with the pathology of schizophrenia to provide a more personalized approach to the patients.

## Data Availability

No datasets were generated or analysed during the current study.

## References

[CR1] Al-Amin MM, Nasir Uddin MM, Mahmud Reza H (2013) Effects of antipsychotics on the inflammatory response system of patients with schizophrenia in peripheral blood mononuclear cell cultures. Clin Psychopharmacol Neurosci 11:144–151. 10.9758/cpn.2013.11.3.144 24465251 10.9758/cpn.2013.11.3.144 PMC3897763

[CR2] Alciati A, Fusi A, D’Arminio Monforte A, Coen M, Ferri A, Mellado C (2001) New-onset delusions and hallucinations in patients infected with HIV. J Psychiatry Neurosci 26:229–234 11394192 PMC1408305

[CR3] Alvarez-Herrera S, Escamilla R, Medina-Contreras O, Saracco R, Flores Y, Hurtado-Alvarado G, Maldonado-García JL, Becerril-Villanueva E, Pérez-Sánchez G, Pavón L (2020) Immunoendocrine Peripheral effects Induced by Atypical Antipsychotics. Front Endocrinol (Lausanne) 11:195. 10.3389/fendo.2020.00195 32373066 10.3389/fendo.2020.00195 PMC7186385

[CR4] Atlas A, Gisslén M, Nordin C, Lindström L, Schwieler L (2007) Acute psychotic symptoms in HIV-1 infected patients are associated with increased levels of kynurenic acid in cerebrospinal fluid. Brain Behav Immun 21:86–91. 10.1016/j.bbi.2006.02.005 16603336 10.1016/j.bbi.2006.02.005

[CR5] Badawy AA (2017) Kynurenine Pathway of Tryptophan Metabolism: Regulatory and functional aspects. Int J Tryptophan Res 10:1178646917691938. 10.1177/1178646917691938 28469468 10.1177/1178646917691938 PMC5398323

[CR6] Barichello T, Simoes LR, Quevedo J, Zhang XY (2019) Microglial activation and psychotic disorders: evidence from pre-clinical and clinical studies. Curr Top Behav Neurosci 44:161–205. 10.1007/7854_2018_81 10.1007/7854_2018_81 30828767

[CR7] Beaulieu JM, Gainetdinov RR (2011) The physiology, signaling, and pharmacology of dopamine receptors. Pharmacol Rev 63:182–217. 10.1124/pr.110.002642 21303898 10.1124/pr.110.002642

[CR8] Behrens MM, Ali SS, Dugan LL (2008) Interleukin-6 mediates the increase in NADPH-oxidase in the ketamine model of schizophrenia. J Neurosci 28:13957–13966. 10.1523/JNEUROSCI.4457-08.2008 19091984 10.1523/JNEUROSCI.4457-08.2008 PMC2752712

[CR9] Bergink V, Gibney SM, Drexhage HA (2014) Autoimmunity, inflammation, and psychosis: a search for peripheral markers. Biol Psychiatry 75:324–331. 10.1016/j.biopsych.2013.09.037 24286760 10.1016/j.biopsych.2013.09.037

[CR10] Bergquist J, Tarkowski A, Ekman R, Ewing A (1994) Discovery of endogenous catecholamines in lymphocytes and evidence for catecholamine regulation of lymphocyte function via an autocrine loop. Proc Natl Acad Sci U S A 91:12912–12916. 10.1073/pnas.91.26.12912 7809145 10.1073/pnas.91.26.12912 PMC45550

[CR11] Bernstein HG, Steiner J, Guest PC, Dobrowolny H, Bogerts B (2015) Glial cells as key players in schizophrenia pathology: recent insights and concepts of therapy. Schizophr Res 161:4–18. 10.1016/j.schres.2014.03.035 24948484 10.1016/j.schres.2014.03.035

[CR12] Brand BA, de Boer JN, Sommer IEC (2021) Estrogens in schizophrenia: progress, current challenges and opportunities. Curr Opin Psychiatry 34:228–237. 10.1097/YCO.0000000000000699 33560022 10.1097/YCO.0000000000000699 PMC8048738

[CR13] Breier A, Su TP, Saunders R, Carson RE, Kolachana BS, de Bartolomeis A, Weinberger DR, Weisenfeld N, Malhotra AK, Eckelman WC, Pickar D (1997) Schizophrenia is associated with elevated amphetamine-induced synaptic dopamine concentrations: evidence from a novel positron emission tomography method. Proc Natl Acad Sci U S A 94:2569–2574. 10.1073/pnas.94.6.2569 9122236 10.1073/pnas.94.6.2569 PMC20129

[CR14] Brisch R, Saniotis A, Wolf R, Bielau H, Bernstein HG, Steiner J, Bogerts B, Braun K, Jankowski Z, Kumaratilake J, Henneberg M, Gos T (2014) The role of dopamine in schizophrenia from a neurobiological and evolutionary perspective: old fashioned, but still in vogue. Front Psychiatry 5:47. 10.3389/fpsyt.2014.00047 24904434 10.3389/fpsyt.2014.00047 PMC4032934

[CR15] Brown AS, Derkits EJ (2010) Prenatal infection and schizophrenia: a review of epidemiologic and translational studies. Am J Psychiatry 167:261–280. 10.1176/appi.ajp.2009.09030361 20123911 10.1176/appi.ajp.2009.09030361 PMC3652286

[CR16] Brydon L, Harrison NA, Walker C, Steptoe A, Critchley HD (2008) Peripheral inflammation is associated with altered substantia nigra activity and psychomotor slowing in humans. Biol Psychiatry 63:1022–1029. 10.1016/j.biopsych.2007.12.007 18242584 10.1016/j.biopsych.2007.12.007 PMC2885493

[CR17] Bushong EA, Martone ME, Jones YZ, Ellisman MH (2002) Protoplasmic astrocytes in CA1 stratum radiatum occupy separate anatomical domains. J Neurosci 22:183–192. 10.1523/JNEUROSCI.22-01-00183.2002 11756501 10.1523/JNEUROSCI.22-01-00183.2002 PMC6757596

[CR18] Cai Y, Kim DJ, Takahashi T, Broadhurst DI, Yan H, Ma S, Rattray NJW, Casanovas-Massana A, Israelow B, Klein J, Lucas C, Mao T, Moore AJ, Muenker MC, Oh JE, Silva J, Wong P, Yale IMPACT Research team, Ko AI, Khan SA, Iwasaki A, Johnson CH (2021) Kynurenic acid may underlie sex-specific immune responses to COVID-19. Sci Signal 14:eabf8483. 10.1126/scisignal.abf8483 10.1126/scisignal.abf8483 PMC8432948 34230210

[CR19] Capuron L, Pagnoni G, Drake DF, Woolwine BJ, Spivey JR, Crowe RJ, Votaw JR, Goodman MM, Miller AH (2012) Dopaminergic mechanisms of reduced basal ganglia responses to hedonic reward during interferon alfa administration. Arch Gen Psychiatry 69:1044–1053. 10.1001/archgenpsychiatry.2011.2094 23026954 10.1001/archgenpsychiatry.2011.2094 PMC3640298

[CR20] Carson MJ, Doose JM, Melchior B, Schmid CD, Ploix CC (2006) CNS immune privilege: hiding in plain sight. Immunol Rev 213:48–65. 10.1111/j.1600-065X.2006.00441.x 16972896 10.1111/j.1600-065X.2006.00441.x PMC2633103

[CR21] Chan AC, Carter PJ (2010) Therapeutic antibodies for autoimmunity and inflammation. Nat Rev Immunol 10:301–316. 10.1038/nri2761 20414204 10.1038/nri2761

[CR22] Chen W (2011) IDO: more than an enzyme. Nat Immunol 12:809–811. 10.1038/ni.2088 21852775 10.1038/ni.2088

[CR23] Chen ML, Tsai TC, Wang LK, Lin YY, Tsai YM, Lee MC, Tsai FM (2012) Risperidone modulates the cytokine and chemokine release of dendritic cells and induces TNF-α-directed cell apoptosis in neutrophils. Int Immunopharmacol 12:197–204. 10.1016/j.intimp.2011.11.011 22154580 10.1016/j.intimp.2011.11.011

[CR24] Chen X, Xiong Z, Li Z, Yang Y, Zheng Z, Li Y, Xie Y, Li Z (2018) Minocycline as adjunct therapy for a male patient with deficit schizophrenia. Neuropsychiatr Dis Treat 14:2697–2701. 10.2147/NDT.S179658 30349268 10.2147/NDT.S179658 PMC6188198

[CR25] Chen L, Zheng WH, Du Y, Li XS, Yu Y, Wang H, Cheng Y (2021) Altered Peripheral Immune profiles in First-Episode, drug-free patients with Schizophrenia: response to antipsychotic medications. Front Med (Lausanne) 8:757655. 10.3389/fmed.2021.757655 34901070 10.3389/fmed.2021.757655 PMC8652082

[CR26] Cho M, Lee TY, Kwak YB, Yoon YB, Kim M, Kwon JS (2019) Adjunctive use of anti-inflammatory drugs for schizophrenia: a meta-analytic investigation of randomized controlled trials. Aust N Z J Psychiatry 53:742–759. 10.1177/0004867419835028 30864461 10.1177/0004867419835028

[CR27] Cole AP, Hoffmeyer E, Chetty SL, Cruz-Cruz J, Hamrick F, Youssef O, Cheshier S, Mitra SS (2020) Microglia in the Brain Tumor Microenvironment. Adv Exp Med Biol 1273:197–208. 10.1007/978-3-030-49270-0_11 33119883 10.1007/978-3-030-49270-0_11

[CR28] Colton CA (2009) Heterogeneity of microglial activation in the innate immune response in the brain. J Neuroimmune Pharmacol 4:399–418. 10.1007/s11481-009-9164-4 19655259 10.1007/s11481-009-9164-4 PMC2773116

[CR29] Cosentino M, Fietta AM, Ferrari M, Rasini E, Bombelli R, Carcano E, Saporiti F, Meloni F, Marino F, Lecchini S (2007) Human CD4 + CD25 + regulatory T cells selectively express tyrosine hydroxylase and contain endogenous catecholamines subserving an autocrine/paracrine inhibitory functional loop. Blood 109:632–642. 10.1182/blood-2006-01-028423 16985181 10.1182/blood-2006-01-028423

[CR30] Coyle JT (2006) Glutamate and schizophrenia: beyond the dopamine hypothesis. Cell Mol Neurobiol 26:363–382. 10.1007/s10571-006-9062-8 10.1007/s10571-006-9062-8 PMC11881825 16773445

[CR31] Cunnington C, Channon KM (2010) Tetrahydrobiopterin: pleiotropic roles in cardiovascular pathophysiology. Heart 96:1872–1877. 10.1136/hrt.2009.180430 20837663 10.1136/hrt.2009.180430

[CR32] Dahan S, Bragazzi NL, Yogev A, Bar-Gad M, Barak V, Amital H, Amital D (2018) The relationship between serum cytokine levels and degree of psychosis in patients with schizophrenia. Psychiatry Res 268:467–472. 10.1016/j.psychres.2018.07.041 30138859 10.1016/j.psychres.2018.07.041

[CR33] Danbolt NC (2001) Glutamate uptake. Prog Neurobiol 65:1–105. 10.1016/s0301-0082(00)00067-8 11369436 10.1016/s0301-0082(00)00067-8

[CR34] Davis KL, Kahn RS, Ko G, Davidson M (1991) Dopamine in schizophrenia: a review and reconceptualization. Am J Psychiatry 148:1474–1486. 10.1176/ajp.148.11.1474 1681750 10.1176/ajp.148.11.1474

[CR35] Delaney S, Fallon B, Alaedini A, Yolken R, Indart A, Feng T, Wang Y, Javitt D (2019) Inflammatory biomarkers in psychosis and clinical high-risk populations. Schizophr Res 206:440–443. 10.1016/j.schres.2018.10.017 30414721 10.1016/j.schres.2018.10.017

[CR36] Diaz FJ, Pérez-Iglesias R, Mata I, Martínez-Garcia O, Vázquez-Barquero JL, de Leon J, Crespo-Facorro B (2010) Possible effects of some antipsychotic drugs on C-reactive protein in a drug-naïve psychotic sample. Schizophr Res 121:207–212. 10.1016/j.schres.2010.06.002 20580206 10.1016/j.schres.2010.06.002

[CR37] Dinan TG, Stanton C, Cryan JF (2013) Psychobiotics: a novel class of psychotropic. Biol Psychiatry 74:720–726. 10.1016/j.biopsych.2013.05.001 23759244 10.1016/j.biopsych.2013.05.001

[CR38] Dunleavy C, Elsworthy RJ, Upthegrove R, Wood SJ, Aldred S (2022) Inflammation in first-episode psychosis: the contribution of inflammatory biomarkers to the emergence of negative symptoms, a systematic review and meta-analysis. Acta Psychiatr Scand 146:6–20. 10.1111/acps.13416 35202480 10.1111/acps.13416 PMC9310618

[CR39] Egerton A, Chaddock CA, Winton-Brown TT, Bloomfield MA, Bhattacharyya S, Allen P, McGuire PK, Howes OD (2013) Presynaptic striatal dopamine dysfunction in people at ultra-high risk for psychosis: findings in a second cohort. Biol Psychiatry 74:106–112. 10.1016/j.biopsych.2012.11.017 23312565 10.1016/j.biopsych.2012.11.017

[CR40] Erhardt S, Blennow K, Nordin C, Skogh E, Lindström LH, Engberg G (2001) Kynurenic acid levels are elevated in the cerebrospinal fluid of patients with schizophrenia. Neurosci Lett 313:96–98. 10.1016/s0304-3940(01)02242-x 11684348 10.1016/s0304-3940(01)02242-x

[CR41] Erhardt S, Mathe JM, Chergui K, Engberg G, Svensson TH (2002) GABA(B) receptor-mediated modulation of the firing pattern of ventral tegmental area dopamine neurons in vivo. Naunyn Schmiedebergs Arch Pharmacol 365:173–180. 10.1007/s00210-001-0519-5 11882912 10.1007/s00210-001-0519-5

[CR42] Erhardt S, Schwieler L, Nilsson L, Linderholm K, Engberg G (2007) The kynurenic acid hypothesis of schizophrenia. Physiol Behav 92:203–209. 10.1016/j.physbeh.2007.05.025 17573079 10.1016/j.physbeh.2007.05.025

[CR43] Estes ML, McAllister AK (2016) Maternal immune activation: implications for neuropsychiatric disorders. Science 353:772–777. 10.1126/science.aag3194 27540164 10.1126/science.aag3194 PMC5650490

[CR44] Farhadian S, Glick LR, Vogels CBF, Thomas J, Chiarella J, Casanovas-Massana A, Zhou J, Odio C, Vijayakumar P, Geng B, Fournier J, Bermejo S, Fauver JR, Alpert T, Wyllie AL, Turcotte C, Steinle M, Paczkowski P, Dela Cruz C, Wilen C, Ko AI, MacKay S, Grubaugh ND, Spudich S, Barakat LA (2020) Acute encephalopathy with elevated CSF inflammatory markers as the initial presentation of COVID-19. BMC Neurol 20:248. 10.1186/s12883-020-01812-2 32552792 10.1186/s12883-020-01812-2 PMC7301053

[CR45] Farokhnia M, Azarkolah A, Adinehfar F, Khodaie-Ardakani MR, Hosseini SM, Yekehtaz H, Tabrizi M, Rezaei F, Salehi B, Sadeghi SM, Moghadam M, Gharibi F, Mirshafiee O, Akhondzadeh S (2013) N-acetylcysteine as an adjunct to risperidone for treatment of negative symptoms in patients with chronic schizophrenia: a randomized, double-blind, placebo-controlled study. Clin Neuropharmacol 36:185–192. 10.1097/WNF.0000000000000001 24201233 10.1097/WNF.0000000000000001

[CR46] Felger JC, Mun J, Kimmel HL, Nye JA, Drake DF, Hernandez CR, Freeman AA, Rye DB, Goodman MM, Howell LL, Miller AH (2013) Chronic interferon-α decreases dopamine 2 receptor binding and striatal dopamine release in association with anhedonia-like behavior in nonhuman primates. Neuropsychopharmacology 38:2179–2187. 10.1038/npp.2013.115 23657438 10.1038/npp.2013.115 PMC3773667

[CR47] Ferrari M, Godio M, Martini S, Callegari C, Cosentino M, Marino F (2022) Inflammatory markers at baseline correlate with subsequent clinical response to quetiapine in patients with bipolar disorder. Hum Psychopharmacol 37:e2854. 10.1002/hup.2854 36069283 10.1002/hup.2854

[CR48] Fujita Y, Yamashita T (2021) Neuroprotective function of microglia in the developing brain. Neuronal Signal 5:NS20200024. 10.1042/NS20200024 33532089 10.1042/NS20200024 PMC7823182

[CR49] Fusar-Poli P, Meyer-Lindenberg A (2013) Striatal presynaptic dopamine in schizophrenia, part I: meta-analysis of dopamine active transporter (DAT) density. Schizophr Bull 39:22–32. 10.1093/schbul/sbr111 22282456 10.1093/schbul/sbr111 PMC3523907

[CR50] Gallego JA, Blanco EA, Husain-Krautter S, Madeline Fagen E, Moreno-Merino P, Del Ojo-Jiménez JA, Ahmed A, Rothstein TL, Lencz T, Malhotra AK (2018) Cytokines in cerebrospinal fluid of patients with schizophrenia spectrum disorders: New data and an updated meta-analysis. Schizophr Res 202:64–71. 10.1016/j.schres.2018.07.019 30025760 10.1016/j.schres.2018.07.019 PMC6564675

[CR51] Gaskill PJ, Carvallo L, Eugenin EA, Berman JW (2012) Characterization and function of the human macrophage dopaminergic system: implications for CNS disease and drug abuse. J Neuroinflammation 9:1–14. 10.1186/1742-2094-9-203 22901451 10.1186/1742-2094-9-203 PMC3488577

[CR52] Geisler S, Gostner JM, Becker K, Ueberall F, Fuchs D (2013) Immune activation and inflammation increase the plasma phenylalanine-to-tyrosine ratio. Pteridines 24:27–31. 10.1515/pterid-2013-0001

[CR53] Ghanizadeh A, Rezaee Z, Dehbozorgi S, Berk M, Akhondzadeh S (2014) Lovastatin for the adjunctive treatment of schizophrenia: a preliminary randomized double-blind placebo-controlled trial. Psychiatry Res 219:431–435. 10.1016/j.psychres.2014.06.039 25017614 10.1016/j.psychres.2014.06.039

[CR54] Ghlichloo I, Gerriets V (2023) Nonsteroidal Anti-Inflammatory Drugs (NSAIDs). In: StatPearls [Internet]. StatPearls Publishing, Treasure Island. 2025 Jan. https://www.ncbi.nlm.nih.gov/books/NBK547742/31613522

[CR55] Ghosh SS, Wang J, Yannie PJ, Ghosh S (2020) Intestinal barrier dysfunction, LPS translocation, and Disease Development. J Endocr Soc 4:bvz039. 10.1210/jendso/bvz039 32099951 10.1210/jendso/bvz039 PMC7033038

[CR56] Girgis RR, Ciarleglio A, Choo T, Haynes G, Bathon JM, Cremers S, Kantrowitz JT, Lieberman JA, Brown AS (2018) A Randomized, Double-Blind, placebo-controlled clinical trial of Tocilizumab, an Interleukin-6 receptor antibody, for residual symptoms in Schizophrenia. Neuropsychopharmacology 43:1317–1323. 10.1038/npp.2017.258 29090685 10.1038/npp.2017.258 PMC5916349

[CR57] Goldsmith DR, Rapaport MH, Miller BJ (2016) A meta-analysis of blood cytokine network alterations in psychiatric patients: comparisons between schizophrenia, bipolar disorder and depression. Mol Psychiatry 21:1696–1709. 10.1038/mp.2016.3 26903267 10.1038/mp.2016.3 PMC6056174

[CR58] Hadar R, Dong L, Del-Valle-Anton L, Guneykaya D, Voget M, Edemann-Callesen H, Schweibold R, Djodari-Irani A, Goetz T, Ewing S, Kettenmann H, Wolf SA, Winter C (2017) Deep brain stimulation during early adolescence prevents microglial alterations in a model of maternal immune activation. Brain Behav Immun 63:71–80. 10.1016/j.bbi.2016.12.003 27939248 10.1016/j.bbi.2016.12.003

[CR59] Haring L, Koido K, Vasar V, Leping V, Zilmer K, Zilmer M, Vasar E (2015) Antipsychotic treatment reduces psychotic symptoms and markers of low-grade inflammation in first episode psychosis patients, but increases their body mass index. Schizophr Res 169:22–29. 10.1016/j.schres.2015.08.027 26364730 10.1016/j.schres.2015.08.027

[CR60] Hou Y, Wu CF, Yang JY, He X, Bi XL, Yu L, Guo T (2006) Effects of clozapine, olanzapine and haloperidol on nitric oxide production by lipopolysaccharide-activated N9 cells. Prog Neuropsychopharmacol Biol Psychiatry 30:1523–1528. 10.1016/j.pnpbp.2006.05.006 16806626 10.1016/j.pnpbp.2006.05.006

[CR61] Howes OD, Kambeitz J, Kim E, Stahl D, Slifstein M, Abi-Dargham A, Kapur S (2012) The nature of dopamine dysfunction in schizophrenia and what this means for treatment: meta-analysis of imaging studies. Arch Gen Psychiatry 69:776–786. 10.1001/archgenpsychiatry.2012.169 22474070 10.1001/archgenpsychiatry.2012.169 PMC3730746

[CR62] Hsuchou H, Pan W, Barnes MJ, Kastin AJ (2009) Leptin receptor mRNA in rat brain astrocytes. Peptides 30:2275–2280. 10.1016/j.peptides.2009.08.023 19747514 10.1016/j.peptides.2009.08.023 PMC2787995

[CR63] Huhn M, Nikolakopoulou A, Schneider-Thoma J, Krause M, Samara M, Peter N, Arndt T, Bäckers L, Rothe P, Cipriani A, Davis J, Salanti G, Leucht S (2019) Comparative efficacy and tolerability of 32 oral antipsychotics for the acute treatment of adults with multi-episode schizophrenia: a systematic review and network meta-analysis. Lancet 394:939–951. 10.1016/S0140-6736(19)31135-3 31303314 10.1016/S0140-6736(19)31135-3 PMC6891890

[CR64] Iliopoulou SM, Tsartsalis S, Kaiser S, Millet P, Tournier BB (2021) Dopamine and neuroinflammation in Schizophrenia–Interpreting the findings from Translocator protein (18 kDa) PET imaging. Neuropsychiatr Dis Treat 17:3345–3357. 10.2147/NDT.S334027 34819729 10.2147/NDT.S334027 PMC8608287

[CR65] Johansson AS, Owe-Larsson B, Asp L, Kocki T, Adler M, Hetta J, Gardner R, Lundkvist GB, Urbanska EM, Karlsson H (2013) Activation of kynurenine pathway in ex vivo fibroblasts from patients with bipolar disorder or schizophrenia: cytokine challenge increases production of 3-hydroxykynurenine. J Psychiatr Res 47:1815–1823. 10.1016/j.jpsychires.2013.08.008 24012176 10.1016/j.jpsychires.2013.08.008

[CR66] Ju S, Shin Y, Han S, Kwon J, Choi TG, Kang I, Kim SS (2023) The gut-brain axis in schizophrenia: the implications of the gut microbiome and SCFA Production. Nutrients 15:4391. 10.3390/nu15204391 10.3390/nu15204391 PMC10610543 37892465

[CR67] Juncal-Ruiz M, Riesco-Dávila L, de la Ortiz-García V, Martínez-Garcia O, Ramírez-Bonilla M, Ocejo-Viñals JG, Leza JC, López-Hoyos M, Crespo-Facorro B (2018) Comparison of the anti-inflammatory effect of aripiprazole and risperidone in 75 drug-naïve first episode psychosis individuals: a 3 months randomized study. Schizophr Res 202:226–233. 10.1016/j.schres.2018.06.039 29941296 10.1016/j.schres.2018.06.039

[CR68] Kato T, Mizoguchi Y, Monji A, Horikawa H, Suzuki SO, Seki Y, Iwaki T, Hashioka S, Kanba S (2008) Inhibitory effects of aripiprazole on interferon-gamma-induced microglial activation via intracellular Ca2 + regulation in vitro. J Neurochem 106:815–825. 10.1111/j.1471-4159.2008.05435.x 18429930 10.1111/j.1471-4159.2008.05435.x

[CR69] Kearns R (2024) Gut-Brain Axis and Neuroinflammation: the role of gut permeability and the Kynurenine Pathway in Neurological disorders. Cell Mol Neurobiol 44:64. 10.1007/s10571-024-01496-z 39377830 10.1007/s10571-024-01496-z PMC11461658

[CR70] Kim HW, Cheon Y, Modi HR, Rapoport SI, Rao JS (2012) Effects of chronic clozapine administration on markers of arachidonic acid cascade and synaptic integrity in rat brain. Psychopharmacology 222:663–674. 10.1007/s00213-012-2671-7 22414961 10.1007/s00213-012-2671-7 PMC3478065

[CR71] Kowalski J, Labuzek K, Herman ZS (2004) Flupentixol and trifluperidol reduce interleukin-1 beta and interleukin-2 release by rat mixed glial and microglial cell cultures. Pol J Pharmacol 56:563–570 15591644

[CR72] Kudlek MS, Mihaljevic-Peles A, Sagud M, Bajs JM, Ganoci L, Grubisin J, Kuzman Rojnic M, Vuksan CB, Bradaš Z, Božina N (2017) Brain-derived neurotrophic factor serum and plasma levels in the treatment of acute schizophrenia with olanzapine or risperidone: 6-week prospective study. Nord J Psychiatry 71:513–520. 10.1080/08039488.2017.1340518 10.1080/08039488.2017.1340518 28671000

[CR73] Kuhlman KR, Robles TF, Dooley LN, Boyle CC, Haydon MD, Bower JE (2018) Within-subject associations between inflammation and features of depression: using the flu vaccine as a mild inflammatory stimulus. Brain Behav Immun 69:540–547. 10.1016/j.bbi.2018.02.001 29458196 10.1016/j.bbi.2018.02.001 PMC5857469

[CR74] Kulaga SS, Miller CWT (2021) Viral respiratory infections and psychosis: a review of the literature and the implications of COVID-19. Neurosci Biobehav Rev 127:520–530. 10.1016/j.neubiorev.2021.05.008 33992695 10.1016/j.neubiorev.2021.05.008 PMC9616688

[CR75] Laan W, Grobbee DE, Selten JP, Heijnen CJ, Kahn RS, Burger H (2010) Adjuvant aspirin therapy reduces symptoms of schizophrenia spectrum disorders: results from a randomized, double-blind, placebo-controlled trial. J Clin Psychiatry 71:520–527. 10.4088/JCP.09m05117yel 20492850 10.4088/JCP.09m05117yel

[CR76] Labuzek K, Kowalski J, Gabryel B, Herman ZS (2005) Chlorpromazine and loxapine reduce interleukin-1β and interleukin-2 release by rat mixed glial and microglial cell cultures. Eur Neuropsychopharmacol 15:23–30. 10.1016/j.euroneuro.2004.04.002 15572270 10.1016/j.euroneuro.2004.04.002

[CR77] Lago-Baldaia I, Fernandes VM, Ackerman SD (2020) More than mortar: glia as architects of nervous system development and disease. Front Cell Dev Biol 8:1527. 10.3389/fcell.2020.611269 10.3389/fcell.2020.611269 PMC7767919 33381506

[CR78] Laugeray A, Launay JM, Callebert J, Surget A, Belzung C, Barone PR (2010) Peripheral and cerebral metabolic abnormalities of the tryptophan-kynurenine pathway in a murine model of major depression. Behav Brain Res 210:84–91. 10.1016/j.bbr.2010.02.014 20153778 10.1016/j.bbr.2010.02.014

[CR79] Lawson MA, Parrott JM, McCusker RH, Dantzer R, Kelley KW, O’Connor JC (2013) Intracerebroventricular administration of lipopolysaccharide induces indoleamine-2,3-dioxygenase-dependent depression-like behaviors. J Neuroinflammation 10:87. 10.1186/1742-2094-10-87 23866724 10.1186/1742-2094-10-87 PMC3733827

[CR80] Liddelow S, Barres B (2015) SnapShot: astrocytes in Health and Disease. Cell 162:1170–1170e1. 10.1016/j.cell.2015.08.029 26317476 10.1016/j.cell.2015.08.029

[CR81] Lim ST, Janaway B, Costello H, Trip A, Price G (2020) Persistent psychotic symptoms following COVID-19 infection. BJPsych Open 6:e105. 10.1192/bjo.2020.76 32696735 10.1192/bjo.2020.76 PMC7477483

[CR82] Linderholm KR, Skogh E, Olsson SK, Dahl ML, Holtze M, Engberg G, Samuelsson M, Erhardt S (2012) Increased levels of kynurenine and kynurenic acid in the CSF of patients with schizophrenia. Schizophr Bull 38:426–432. 10.1093/schbul/sbq086 20729465 10.1093/schbul/sbq086 PMC3329991

[CR83] Liu L, Jia F, Yuan G, Chen Z, Yao J, Li H, Fang C (2010) Tyrosine hydroxylase, interleukin-1beta and tumor necrosis factor-alpha are overexpressed in peripheral blood mononuclear cells from schizophrenia patients as determined by semi-quantitative analysis. Psychiatry Res 176:1–7. 10.1016/j.psychres.2008.10.024 20067853 10.1016/j.psychres.2008.10.024

[CR84] Lorrain DS, Baccei CS, Bristow LJ, Anderson JJ, Varney MA (2003) Effects of ketamine and N-methyl-D-aspartate on glutamate and dopamine release in the rat prefrontal cortex: modulation by a group II selective metabotropic glutamate receptor agonist LY379268. Neuroscience 117:697–706. 10.1016/s0306-4522(02)00652-8 12617973 10.1016/s0306-4522(02)00652-8

[CR85] Lurie DI (2018) An Integrative Approach to Neuroinflammation in Psychiatric disorders and Neuropathic Pain. J Exp Neurosci 12:1179069518793639. 10.1177/1179069518793639 30127639 10.1177/1179069518793639 PMC6090491

[CR86] MacDowell KS, García-Bueno B, Madrigal JL, Parellada M, Arango C, Micó JA, Leza JC (2013) Risperidone normalizes increased inflammatory parameters and restores anti-inflammatory pathways in a model of neuroinflammation. Int J Neuropsychopharmacol 16:121–135. 10.1017/S1461145711001775 22176740 10.1017/S1461145711001775

[CR87] Mattei D, Schweibold R, Wolf SA (2015) Brain in flames - animal models of psychosis: utility and limitations. Neuropsychiatr Dis Treat 11:1313–1329. 10.2147/NDT.S65564 26064050 10.2147/NDT.S65564 PMC4455860

[CR88] McGrath J, Saha S, Chant D, Welham J (2008) Schizophrenia: a concise overview of incidence, prevalence, and mortality. Epidemiol Rev 30:67–76. 10.1093/epirev/mxn001 18480098 10.1093/epirev/mxn001

[CR89] Merritt K, McGuire PK, Egerton A, 1H-MRS in Schizophrenia Investigators, Aleman A, Block W, Bloemen OJN, Borgan F, Bustillo JR, Capizzano AA, Coughlin JM, De la Fuente-Sandoval C, Demjaha A, Dempster K, Do KQ, Du F, Falkai P, Galinska-Skok B, Gallinat J, Gasparovic C, Ginestet CE, Goto N, Graff-Guerrero A, Ho BC, Howes OD, Jauhar S, Jeon P, Kato T, Kaufmann CA, Kegeles LS, Keshavan M, Kim SY, Kunugi H, Lauriello J, Liemburg EJ, Mcilwain ME, Modinos G, Mouchlianitis ED, Nakamura J, Nenadic I, Öngür D, Ota M, Palaniyappan L, Pantelis C, Plitman E, Posporelis S, Purdon SE, Reichenbach JR, Renshaw PF, Russell BR, Sawa A, Schaefer M, Shungu DC, Smesny S, Stanley JA, Stone JM, Szulc A, Taylor R, Thakkar K, Théberge J, Tibbo PG, van Amelsvoort T, Walecki J, Williamson PC, Wood SJ, Xin L, Yamasue H (2021) Association of Age, Antipsychotic Medication, and Symptom Severity in Schizophrenia with Proton magnetic resonance spectroscopy brain glutamate level: a mega-analysis of individual participant-Level Data. JAMA Psychiatry 78:667–681. 10.1001/jamapsychiatry.2021.0380 33881460 10.1001/jamapsychiatry.2021.0380 PMC8060889

[CR90] Miller BJ, Buckley P, Seabolt W, Mellor A, Kirkpatrick B (2011) Meta-analysis of cytokine alterations in schizophrenia: clinical status and antipsychotic effects. Biol Psychiatry 70:663–671. 10.1016/j.biopsych.2011.04.013 21641581 10.1016/j.biopsych.2011.04.013 PMC4071300

[CR91] Miller BJ, Dias JK, Lemos HP, Buckley PF (2016) An open-label, pilot trial of adjunctive tocilizumab in schizophrenia. J Clin Psychiatry 77:275–276. 10.4088/JCP.15l09920 26930525 10.4088/JCP.15l09920

[CR92] Mittal R, Debs LH, Patel AP, Nguyen D, Patel K, O’Connor G, Grati M, Mittal J, Yan D, Eshraghi AA, Deo SK, Daunert S, Liu XZ (2017) Neurotransmitters: the critical modulators regulating gut-brain Axis. J Cell Physiol 232:2359–2372. 10.1002/jcp.25518 27512962 10.1002/jcp.25518 PMC5772764

[CR93] Mizoguchi H, Takuma K, Fukakusa A, Ito Y, Nakatani A, Ibi D, Kim HC, Yamada K (2008) Improvement by minocycline of methamphetamine-induced impairment of recognition memory in mice. Psychopharmacology 196:233–241. 10.1007/s00213-007-0955-0 17909751 10.1007/s00213-007-0955-0

[CR94] Moghaddam B, Javitt D (2012) From revolution to evolution: the glutamate hypothesis of schizophrenia and its implication for treatment. Neuropsychopharmacology 37:4–15. 10.1038/npp.2011.181 21956446 10.1038/npp.2011.181 PMC3238069

[CR95] Möller M, Swanepoel T, Harvey BH (2015) Neurodevelopmental animal models reveal the Convergent Role of Neurotransmitter Systems, inflammation, and oxidative stress as biomarkers of Schizophrenia: implications for Novel Drug Development. ACS Chem Neurosci 6:987–1016. 10.1021/cn5003368 25794269 10.1021/cn5003368

[CR96] Moreno-Küstner B, Martín C, Pastor L (2018) Prevalence of psychotic disorders and its association with methodological issues. A systematic review and meta-analyses. PLoS ONE 13:e0195687. 10.1371/journal.pone.0195687 29649252 10.1371/journal.pone.0195687 PMC5896987

[CR97] Muench J, Hamer AM (2010) Adverse effects of antipsychotic medications. Am Fam Physician 81:617–622 20187598

[CR98] Müller N, Riedel M, Scheppach C, Brandstätter B, Sokullu S, Krampe K, Ulmschneider M, Engel RR, Möller HJ, Schwarz MJ (2002) Beneficial antipsychotic effects of celecoxib add-on therapy compared to risperidone alone in schizophrenia. Am J Psychiatry 159:1029–1034. 10.1176/appi.ajp.159.6.1029 10.1176/appi.ajp.159.6.1029 12042193

[CR99] Muneer A (2020) Kynurenine pathway of tryptophan metabolism in neuropsychiatric disorders: pathophysiologic and therapeutic considerations. Clin Psychopharmacol Neurosci 18:507–526. 10.9758/cpn.2020.18.4.507 33124585 10.9758/cpn.2020.18.4.507 PMC7609208

[CR100] Murr C, Widner B, Wirleitner B, Fuchs D (2002) Neopterin as a marker for immune system activation. Curr Drug Metab 3:175–187. 10.2174/1389200024605082 12003349 10.2174/1389200024605082

[CR101] Nakanishi S (1992) Molecular diversity of glutamate receptors and implications for brain function. Science 258:597–603. 10.1126/science.1329206 1329206 10.1126/science.1329206

[CR102] Neurauter G, Schröcksnadel K, Scholl-Bürgi S, Sperner-Unterweger B, Schubert C, Ledochowski M, Fuchs D (2008) Chronic immune stimulation correlates with reduced phenylalanine turnover. Curr Drug Metab 9:622–627. 10.2174/138920008785821738 18781914 10.2174/138920008785821738

[CR103] Nimmerjahn A, Kirchhoff F, Helmchen F (2005) Resting microglial cells are highly dynamic surveillants of brain parenchyma in vivo. Science 308:1314–1318. 10.1126/science.1110647 15831717 10.1126/science.1110647

[CR104] Nitta M, Kishimoto T, Müller N, Weiser M, Davidson M, Kane JM, Correll CU (2013) Adjunctive use of nonsteroidal anti-inflammatory drugs for schizophrenia: a meta-analytic investigation of randomized controlled trials. Schizophr Bull 39:1230–1241. 10.1093/schbul/sbt070 23720576 10.1093/schbul/sbt070 PMC3796088

[CR105] Nomura I, Kishi T, Ikuta T, Iwata N (2018) Statin add-on therapy in the antipsychotic treatment of schizophrenia: a meta-analysis. Psychiatry Res 260:41–47. 10.1016/j.psychres.2017.11.033 29172097 10.1016/j.psychres.2017.11.033

[CR106] Noto C, Ota VK, Gouvea ES, Rizzo LB, Spindola LM, Honda PH, Cordeiro Q, Belangero SI, Bressan RA, Gadelha A, Maes M, Brietzke E (2014) Effects of risperidone on cytokine profile in drug-naïve first-episode psychosis. Int J Neuropsychopharmacol 18:pyu042. 10.1093/ijnp/pyu042 25522386 10.1093/ijnp/pyu042 PMC4360233

[CR107] Opsteen S, Files JK, Fram T, Erdmann N (2023) The role of immune activation and antigen persistence in acute and long COVID. J Investig Med 71:545–562. 10.1177/10815589231158041 36879504 10.1177/10815589231158041 PMC9996119

[CR108] Palacio JR, Markert UR, Martínez P (2011) Anti-inflammatory properties of N-acetylcysteine on lipopolysaccharide-activated macrophages. Inflamm Res 60(7):695–704. 10.1007/s00011-011-0323-8 21424515 10.1007/s00011-011-0323-8

[CR109] Pan W, Hsuchou H, Jayaram B, Khan RS, Huang EY, Wu X, Chen C, Kastin AJ (2012) Leptin action on nonneuronal cells in the CNS: potential clinical applications. Ann N Y Acad Sci 1264:64–71. 10.1111/j.1749-6632.2012.06472.x 22530983 10.1111/j.1749-6632.2012.06472.x PMC3407332

[CR110] Paoletti P, Bellone C, Zhou Q (2013) NMDA receptor subunit diversity: impact on receptor properties, synaptic plasticity and disease. Nat Rev Neurosci 14:383–400. 10.1038/nrn3504 23686171 10.1038/nrn3504

[CR111] Pardo-de-Santayana G, Juncal-Ruiz M, Vázquez-Bourgon J, Riesco-Dávila L, de la Ortiz-Garcia V, Pelayo-Terán JM, López-Hoyos M, Crespo-Facorro B (2021) Active psychosis and pro-inflammatory cytokines in first-episode of psychosis. J Psychiatr Res 134:150–157. 10.1016/j.jpsychires.2020.12.060 33385633 10.1016/j.jpsychires.2020.12.060

[CR112] Patel KR, Cherian J, Gohil K, Atkinson D (2014) Schizophrenia: overview and treatment options. P T 39:638–645 25210417 PMC4159061

[CR113] Plitman E, Iwata Y, Caravaggio F, Nakajima S, Chung JK, Gerretsen P, Kim J, Takeuchi H, Chakravarty MM, Remington G, Graff-Guerrero A (2017) Kynurenic acid in schizophrenia: a systematic review and meta-analysis. Schizophr Bull 43:764–777. 10.1093/schbul/sbw221 28187219 10.1093/schbul/sbw221 PMC5472151

[CR114] Potter ED, Ling ZD, Carvey PM (1999) Cytokine-induced conversion of mesencephalic-derived progenitor cells into dopamine neurons. Cell Tissue Res 296:235–246. 10.1007/s004410051285 10382268 10.1007/s004410051285

[CR115] Pratt L, Ni L, Ponzio NM, Jonakait GM (2013) Maternal inflammation promotes fetal microglial activation and increased cholinergic expression in the fetal basal forebrain: role of interleukin-6. Pediatr Res 74:393–401. 10.1038/pr.2013.126 23877071 10.1038/pr.2013.126

[CR116] Qiu YH, Peng YP, Jiang JM, Wang JJ (2004) Expression of tyrosine hydroxylase in lymphocytes and effect of endogenous catecholamines on lymphocyte function. Neuroimmunomodulation 11:75–83. 10.1159/000075316 14758053 10.1159/000075316

[CR117] Racki V, Marcelic M, Stimac I, Petric D, Kucic N (2021) Effects of Haloperidol, Risperidone, and Aripiprazole on the Immunometabolic properties of BV-2 microglial cells. Int J Mol Sci 22:4399. 10.3390/ijms22094399 33922377 10.3390/ijms22094399 PMC8122792

[CR118] Razumovitch JA, Semenkova GN, Fuchs D, Cherenkevich SN (2003) Influence of neopterin on the generation of reactive oxygen species in human neutrophils. FEBS Lett 549:83–86. 10.1016/s0014-5793(03)00796-8 12914930 10.1016/s0014-5793(03)00796-8

[CR119] Reinfeld S, Cáceda R, Gil R, Strom H, Chacko M (2021) Can new onset psychosis occur after mRNA based COVID-19 vaccine administration? A case report. Psychiatry Res 304:114165. 10.1016/j.psychres.2021.114165 34388513 10.1016/j.psychres.2021.114165 PMC8349391

[CR120] Rentero D, Juanes A, Losada CP, Álvarez S, Parra A, Santana V, Martí I, Urricelqui J (2021) New-onset psychosis in COVID-19 pandemic: a case series in Madrid. Psychiatry Res 290:113097. 10.1016/j.psychres.2020.113097 10.1016/j.psychres.2020.113097 PMC7217785 32480119

[CR121] Ribeiro BM, do Carmo MR, Freire RS, Rocha NF, Borella VC, de Menezes AT, Monte AS, Gomes PX, de Sousa FC, Vale ML, de Lucena DF, Gama CS, Macêdo D (2013) Evidences for a progressive microglial activation and increase in iNOS expression in rats submitted to a neurodevelopmental model of schizophrenia: reversal by clozapine. Schizophr Res 151:12–19. 10.1016/j.schres.2013.10.040 24257517 10.1016/j.schres.2013.10.040

[CR122] Richardson MA, Read LL, Clelland CLT, Reilly MA, Chao HM, Guynn RW, Suckow RF, Clelland JD (2005) Evidence for a tetrahydrobiopterin deficit in schizophrenia. Neuropsychobiology 52:190–201. 10.1159/000089002 16244500 10.1159/000089002

[CR123] Richardson MA, Read LL, Reilly MA, Clelland JD, Clelland CLT (2007) Analysis of plasma biopterin levels in psychiatric disorders suggests a common BH4 deficit in schizophrenia and schizoaffective disorder. Neurochem Res 32:107–113. 10.1007/s11064-006-9233-5 17160504 10.1007/s11064-006-9233-5

[CR124] Romeo B, Brunet-Lecomte M, Martelli C, Benyamina A (2018) Kinetics of cytokine levels during antipsychotic treatment in schizophrenia: a meta-analysis. Int J Neuropsychopharmacol 21:828–836. 10.1093/ijnp/pyy062 30016466 10.1093/ijnp/pyy062 PMC6119290

[CR125] Romeo B, Rari E, Mazari A, Toullec A, Martelli C, Benyamina A (2021) First-episode psychosis following vaccination against yellow fever: a case report. Encephale 47:630–631. 10.1016/j.encep.2020.09.009 33541715 10.1016/j.encep.2020.09.009

[CR126] Rubio MD, Drummond JB, Meador-Woodruff JH (2012) Glutamate receptor abnormalities in schizophrenia: implications for innovative treatments. Biomol Ther 20:1–18. 10.4062/biomolther.2012.20.1.001 10.4062/biomolther.2012.20.1.001 PMC3792192 24116269

[CR127] Ryan RM, Ingram SL, Scimemi A (2021) Regulation of glutamate, GABA and dopamine transporter uptake, surface mobility and expression. Front Cell Neurosci 15:670346. 10.3389/fncel.2021.670346 33927596 10.3389/fncel.2021.670346 PMC8076567

[CR128] Salter MW, Beggs S (2014) Sublime microglia: expanding roles for the guardians of the CNS. Cell 158:15–24. 10.1016/j.cell.2014.06.008 24995975 10.1016/j.cell.2014.06.008

[CR129] Salter MW, Stevens B (2017) Microglia emerge as central players in brain disease. Nat Med 23:1018–1102. 10.1038/nm.4397 28886007 10.1038/nm.4397

[CR130] Sarkar A, Lehto SM, Harty S, Dinan TG, Cryan JF, Burnet PWJ (2016) Psychobiotics and the manipulation of Bacteria-gut-brain signals. Trends Neurosci 39:763–781. 10.1016/j.tins.2016.09.002 27793434 10.1016/j.tins.2016.09.002 PMC5102282

[CR131] Schwarcz R, Bruno JP, Muchowski PJ, Wu HQ (2012) Kynurenines in the mammalian brain: when physiology meets pathology. Nat Rev Neurosci 13:465–477. 10.1038/nrn3257 22678511 10.1038/nrn3257 PMC3681811

[CR132] Shah UH, González-Maeso J (2019) Serotonin and glutamate interactions in preclinical schizophrenia models. Acschemneuro 10:3068–3077 10.1021/acschemneuro.9b00044 PMC12502096 30807107

[CR133] Shuto H, Kataoka Y, Horikawa T, Fujihara N, Oishi R (1997) Repeated interferon-α administration inhibits dopaminergic neural activity in the mouse brain. Brain Res 747:348–351. 10.1016/S0006-8993(96)01371-6 9046014 10.1016/s0006-8993(96)01371-6

[CR134] Sobiś J, Rykaczewska-Czerwińska M, Świętochowska E, Gorczyca P (2015) Therapeutic effect of aripiprazole in chronic schizophrenia is accompanied by anti-inflammatory activity. Pharmacol Rep 67:353–359. 10.1016/j.pharep.2014.09.007 25712663 10.1016/j.pharep.2014.09.007

[CR135] Sofroniew MV, Vinters HV (2010) Astrocytes: biology and pathology. Acta Neuropathol 119:7–35. 10.1007/s00401-009-0619-8 20012068 10.1007/s00401-009-0619-8 PMC2799634

[CR136] Solmi M, Veronese N, Thapa N, Facchini S, Stubbs B, Fornaro M, Carvalho AF, Correll CU (2017) Systematic review and meta-analysis of the efficacy and safety of minocycline in schizophrenia. CNS Spectr 22:415–426. 10.1017/S1092852916000638 28181901 10.1017/S1092852916000638

[CR137] Sommer IE, de Witte L, Begemann M, Kahn RS (2011) Nonsteroidal anti-inflammatory drugs in schizophrenia: ready for practice or a good start? A meta-analysis. J Clin Psychiatry 73:414–419. 10.4088/JCP.10r06823 22225599 10.4088/JCP.10r06823

[CR138] Sommer IE, van Westrhenen R, Begemann MJ, de Witte LD, Leucht S, Kahn RS (2014) Efficacy of anti-inflammatory agents to improve symptoms in patients with schizophrenia: an update. Schizophr Bull 40:181–191. 10.1093/schbul/sbt139 24106335 10.1093/schbul/sbt139 PMC3885306

[CR139] Song C, Merali Z, Anisman H (1999) Variations of nucleus accumbens dopamine and serotonin following systemic interleukin-1, interleukin-2 or interleukin-6 treatment. Neuroscience 88:823–836. 10.1016/S0306-4522(98)00271-1 10363820 10.1016/s0306-4522(98)00271-1

[CR140] Stahl SM (2007) Beyond the dopamine hypothesis to the NMDA glutamate receptor hypofunction hypothesis of schizophrenia. CNS Spectr 12:265–268. 10.1017/S1092852900021015 17426663 10.1017/s1092852900021015

[CR141] Stojanovic A, Martorell L, Montalvo I, Ortega L, Monseny R, Vilella E, Labad J (2014) Increased serum interleukin-6 levels in early stages of psychosis: associations with at-risk mental states and the severity of psychotic symptoms. Psychoneuroendocrinology 41:23–32. 10.1016/j.psyneuen.2013.12.005 24495605 10.1016/j.psyneuen.2013.12.005

[CR142] Sullivan PF, Kendler KS, Neale MC (2003) Schizophrenia as a complex trait: evidence from a meta-analysis of twin studies. Arch Gen Psychiatry 60:1187–1192. 10.1001/archpsyc.60.12.1187 10.1001/archpsyc.60.12.1187 14662550

[CR143] Takahashi T, Ellingson MK, Wong P, Israelow B, Lucas C, Klein J, Silva J, Mao T, Oh JE, Tokuyama M, Lu P, Venkataraman A, Park A, Liu F, Meir A, Sun J, Wang EY, Casanovas-Massana A, Wyllie AL, Vogels CBF, Earnest R, Lapidus S, Ott IM, Moore AJ, Yale IMPACT, Research Team, Shaw A, Fournier JB, Odio CD, Farhadian S, Dela Cruz C, Grubaugh ND, Schulz WL, Ring AM, Ko AI, Omer SB, Iwasaki A (2020) Sex differences in immune responses that underlie COVID-19 disease outcomes. Nature 588(7837):315–320. 10.1038/s41586-020-2700-3 32846427 10.1038/s41586-020-2700-3 PMC7725931

[CR144] Tan YL, Yuan Y, Tian L (2020) Microglial regional heterogeneity and its role in the brain. Mol Psychiatry 25:351–367. 10.1038/s41380-019-0609-8 31772305 10.1038/s41380-019-0609-8 PMC6974435

[CR145] Tardiolo G, Bramanti P, Mazzon E (2018) Overview on the effects of *N*-Acetylcysteine in neurodegenerative diseases. Molecules 23:3305. 10.3390/molecules23123305 30551603 10.3390/molecules23123305 PMC6320789

[CR146] Tharoor H, Mara S, Gopal S (2018) Role of novel dietary supplement N-acetyl cysteine in treating negative symptoms in schizophrenia: a 6-month follow-up study. Indian J Psychol Med 40:139–142. 10.4103/IJPSYM.IJPSYM_322_17 29962570 10.4103/IJPSYM.IJPSYM_322_17 PMC6009003

[CR147] Theleritis C, Stefanou MI, Demetriou M, Alevyzakis E, Triantafyllou K, Smyrnis N, Spandidos DA, Rizos E (2024) Association of gut dysbiosis with first episode psychosis (Review). Mol Med Rep 30:130. 10.3892/mmr.2024.13254 10.3892/mmr.2024.13254 PMC11148526 38785152

[CR148] Tutakhail A, Boulet L, Khabil S, Nazari QA, Hamid H, Coudoré F (2020) Neuropathology of kynurenine pathway of tryptophan metabolism. Curr Pharmacol Rep 6:8–23. 10.1007/s40495-019-00208-2

[CR149] Uno Y, Coyle JT (2019) Glutamate hypothesis in schizophrenia. Psychiatry Clin Neurosci 73:204–215. 10.1111/pcn.12823 30666759 10.1111/pcn.12823

[CR150] Upthegrove R, Manzanares-Teson N, Barnes NM (2014) Cytokine function in medication-naive first episode psychosis: a systematic review and meta-analysis. Schizophr Res 155:101–108. 10.1016/j.schres.2014.03.005 24704219 10.1016/j.schres.2014.03.005

[CR151] van Kesteren CF, Gremmels H, de Witte LD, Hol EM, Van Gool AR, Falkai PG, Kahn RS, Sommer IE (2017) Immune involvement in the pathogenesis of schizophrenia: a meta-analysis on postmortem brain studies. Transl Psychiatry 7:e1075. 10.1038/tp.2017.4 28350400 10.1038/tp.2017.4 PMC5404615

[CR152] Vécsei L, Szalárdy L, Fülöp F, Toldi J (2013) Kynurenines in the CNS: recent advances and new questions. Nat Rev Drug Discov 12:64–82. 10.1038/nrd3793 10.1038/nrd3793 23237916

[CR153] Vidal PM, Pacheco R (2020) The Cross-talk between the dopaminergic and the Immune System involved in Schizophrenia. Front Pharmacol 11:394. 10.3389/fphar.2020.00394 32296337 10.3389/fphar.2020.00394 PMC7137825

[CR154] Walker AK, Budac DP, Bisulco S, Lee AW, Smith RA, Beenders B, Kelley KW, Dantzer R (2013) NMDA receptor blockade by ketamine abrogates lipopolysaccharide-induced depressive-like behavior in C57BL/6J mice. Neuropsychopharmacology 38:1609–1616. 10.1038/npp.2013.71 23511700 10.1038/npp.2013.71 PMC3717543

[CR155] Wang AK, Miller BJ (2018) Meta-analysis of Cerebrospinal Fluid Cytokine and Tryptophan Catabolite alterations in Psychiatric patients: comparisons between Schizophrenia, bipolar disorder, and Depression. Schizophr Bull 44:75–83. 10.1093/schbul/sbx035 28338954 10.1093/schbul/sbx035 PMC5768046

[CR156] Weickert T, Jacomb I, Lenroot R, Lappin J, Weinberg D, Brooks W, Brown D, Pellen D, Kindler J, Mohan A, Wakefield D, Lloyd A, Stanton C, O’Donnell M, Liu D, Galletly C, Weickert CS (2019) S33. Reduction in peripheral c-reactive protein levels with canakinumab administration is related to reduced positive symptom severity in patients with schizophrenia and inflammation. Schizophr Bull 45(Suppl 2):S318. 10.1093/schbul/sbz020.578

[CR157] Wirleitner B, Reider D, Ebner S, Böck G, Widner B, Jaeger M, Schennach H, Romani N, Fuchs D (2002) Monocyte-derived dendritic cells release neopterin. J Leukoc Biol 72:1148–1153. 10.1189/jlb.72.6.1148 12488496

[CR158] Wong SS, Oshansky CM, Guo XJ, Ralston J, Wood T, Seeds R, Newbern C, Waite B, Reynolds G, Widdowson MA, Huang QS, Webby RJ, Thomas PG, SHIVERS Investigation Team (2018) Severe influenza is characterized by prolonged Immune activation: results from the SHIVERS Cohort Study. J Infect Dis 217:245–256. 10.1093/infdis/jix571 29112724 10.1093/infdis/jix571 PMC7335675

[CR159] Wu HQ, Okuyama M, Kajii Y, Pocivavsek A, Bruno JP, Schwarcz R (2014) Targeting kynurenine aminotransferase II in psychiatric diseases: promising effects of an orally active enzyme inhibitor. Schizophr Bull 40 Suppl 2(Suppl 2):S152–S158. 10.1093/schbul/sbt157 10.1093/schbul/sbt157 PMC3934402 24562494

[CR160] Xiang S, Ji JL, Li S, Cao XP, Xu W, Tan L, Tan CC (2022) Efficacy and safety of Probiotics for the treatment of Alzheimer’s disease, mild cognitive impairment, and Parkinson’s Disease: a systematic review and Meta-analysis. Front Aging Neurosci 14:730036. 10.3389/fnagi.2022.730036 35185522 10.3389/fnagi.2022.730036 PMC8851038

[CR161] Xiao Y, Sharma MM, Thiruvalluru RK, Gimbrone C, Weissman MM, Olfson M, Keyes KM, Pathak J (2022) Trends in psychiatric diagnoses by COVID-19 infection and hospitalization among patients with and without recent clinical psychiatric diagnoses in New York City from March 2020 to August 2021. Transl Psychiatry 12:492. 10.1038/s41398-022-02255-8 36414624 10.1038/s41398-022-02255-8 PMC9681844

[CR162] Yolland CO, Hanratty D, Neill E, Rossell SL, Berk M, Dean OM, Castle DJ, Tan EJ, Phillipou A, Harris AW, Barreiros AR, Hansen A, Siskind D (2020) Meta-analysis of randomised controlled trials with *N*-acetylcysteine in the treatment of schizophrenia. Aust N Z J Psychiatry 54:453–466. 10.1177/0004867419893439 31826654 10.1177/0004867419893439

[CR163] Zamanian JL, Xu L, Foo LC, Nouri N, Zhou L, Giffard RG, Barres BA (2012) Genomic analysis of reactive astrogliosis. J Neurosci 32:6391–6410. 10.1523/JNEUROSCI.6221-11.2012 22553043 10.1523/JNEUROSCI.6221-11.2012 PMC3480225

[CR164] Zamanpoor M (2020) Schizophrenia in a genomic era: a review from the pathogenesis, genetic and environmental etiology to diagnosis and treatment insights. Psychiatr Genet 30:1–9. 10.1097/YPG.0000000000000245 31764709 10.1097/YPG.0000000000000245

[CR165] Zhang Y, Shi H, Yang G, Yang Y, Li W, Song M, Shao M, Su X, Lv L (2021) Associations between expression of indoleamine 2, 3-dioxygenase enzyme and inflammatory cytokines in patients with first-episode drug-naive Schizophrenia. Transl Psychiatry 11:595. 10.1038/s41398-021-01688-x 34802039 10.1038/s41398-021-01688-x PMC8606005

[CR166] Zhao T, Tang S, Gao X, Li J, Hao R, Chen H, Huang G (2023) Association of serum brain-derived neurotrophic factor level and early response to antipsychotic drug in first-episode patients with schizophrenia. Int J Methods Psychiatr Res 33:e1982. 10.1002/mpr.1982 37485797 10.1002/mpr.1982 PMC10804348

[CR167] Zhao X, Zhu W, Bu Y, Li J, Hao Y, Bi Y (2024) Effects of 6-week olanzapine treatment on serum IL-2, IL-4, IL-8, IL-10, and TNF-α levels in drug-naive individuals with first-episode schizophrenia. BMC Psychiatry 24:703. 10.1186/s12888-024-06163-7 39425118 10.1186/s12888-024-06163-7 PMC11490170

[CR168] Zheng LT, Hwang J, Ock J, Lee MG, Lee WH, Suk K (2008) The antipsychotic spiperone attenuates inflammatory response in cultured microglia via the reduction of proinflammatory cytokine expression and nitric oxide production. J Neurochem 107:1225–1235. 10.1111/j.1471-4159.2008.05675.x 18786164 10.1111/j.1471-4159.2008.05675.x

[CR169] Zheng P, Zeng B, Liu M, Chen J, Pan J, Han Y, Liu Y, Cheng K, Zhou C, Wang H, Zhou X, Gui S, Perry SW, Wong ML, Licinio J, Wei H, Xie P (2019) The gut microbiome from patients with schizophrenia modulates the glutamate-glutamine-GABA cycle and schizophrenia-relevant behaviors in mice. Sci Adv 5:8317. 10.1126/sciadv.aau8317 10.1126/sciadv.aau8317 PMC6365110 30775438

[CR170] Zhu S, Stein RA, Yoshioka C, Lee CH, Goehring A, Mchaourab HS, Gouaux E (2016) Mechanism of NMDA receptor inhibition and activation. Cell 165:704–714. 10.1016/j.cell.2016.03.028 27062927 10.1016/j.cell.2016.03.028 PMC4914038

